# The conserved C2 phospholipid‐binding domain in Delta contributes to robust Notch signalling

**DOI:** 10.15252/embr.202152729

**Published:** 2021-08-04

**Authors:** Torcato Martins, Yao Meng, Boguslawa Korona, Richard Suckling, Steven Johnson, Penny A Handford, Susan M Lea, Sarah J Bray

**Affiliations:** ^1^ Department of Physiology Development and Neuroscience University of Cambridge Cambridge UK; ^2^ Department of Biochemistry University of Oxford Oxford UK; ^3^ Sir William Dunn School of Pathology University of Oxford Oxford UK; ^4^ Present address: Center for Structural Biology CCR, NCI Fort Dettrick Frederick MD 21701 USA

**Keywords:** C2 domain, Delta, *Drosophila*, *Notch* ligands, phospholipid, Development, Signal Transduction, Structural Biology

## Abstract

Accurate Notch signalling is critical for development and homeostasis. Fine‐tuning of Notch–ligand interactions has substantial impact on signalling outputs. Recent structural studies have identified a conserved N‐terminal C2 domain in human Notch ligands which confers phospholipid binding *in vitro*. Here, we show that *Drosophila* ligands Delta and Serrate adopt the same C2 domain structure with analogous variations in the loop regions, including the so‐called β1‐2 loop that is involved in phospholipid binding. Mutations in the β1‐2 loop of the Delta C2 domain retain Notch binding but have impaired ability to interact with phospholipids *in vitro*. To investigate its role *in vivo*, we deleted five residues within the β1‐2 loop of endogenous Delta. Strikingly, this change compromises ligand function. The modified Delta enhances phenotypes produced by Delta loss‐of‐function alleles and suppresses that of Notch alleles. As the modified protein is present on the cell surface in normal amounts, these results argue that C2 domain phospholipid binding is necessary for robust signalling *in vivo* fine‐tuning the balance of trans and cis ligand–receptor interactions.

## Graphical abstract

Engineered mutations in the conserved N‐terminal C2 domains of endogenous Delta and Serrate show that perturbing one of the protruding loops conferring phospholipid binding leads to defective Notch signalling *in vivo*, confirming its contribution to normal ligand activity.

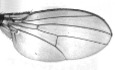

## Introduction

The Notch signalling pathway is highly conserved and plays key roles in many aspects of development and homeostasis (Bray, 2016). Aberrant Notch signalling results in a number of inherited diseases and is associated with various cancers and other acquired disorders (Mašek & Andersson, 2017; Nowell & Radtke, 2017; Siebel & Lendahl, 2017; Monticone & Miele, 2021). As both the Notch receptors and the ligands are single‐pass type I transmembrane proteins, signalling is initiated by direct protein–protein contact between adjacent cells, which may occur in some instances via long cell processes such as cytonemes (De Joussineau *et al*, 2003; Cohen *et al*, 2010; Huang & Kornberg, 2015; Hunter *et al*, 2019; Boukhatmi *et al*, 2020). Canonical Notch signalling involves a simple cascade, whereby ligand binding induces successive cleavages to release the Notch intracellular domain (NICD) which translocates to the nucleus and directly regulates gene expression with its binding partners (Kovall, 2008; Kovall & Blacklow, 2010; Bray, 2016; Kovall *et al*, 2017). One challenge is to understand how this simple core mechanism is modulated to ensure appropriate spatio‐temporal regulation of the pathway. Mechanisms that fine‐tune the ligand–receptor interactions are likely to make important contributions.

All Notch ligands have a similar architecture, with an extracellular domain consisting of multiple (7, 8, or 16) epidermal growth factor (EGF) repeats, a so‐called Delta/Serrate/Lag‐2 (DSL) domain and a highly conserved N‐terminal region (Bray, 2006; D’Souza *et al*, 2008; Kopan & Ilagan, 2009; Kovall & Blacklow, 2010). Receptor binding involves the N‐terminal portion including the DSL and N‐terminal domains (Cordle *et al*, 2008; Luca *et al*, 2015, 2017). Structural studies of the N‐terminal region from human Delta and Jagged ligands revealed that it adopts a conformation characteristic of a phospholipid‐binding C2 domain (Chillakuri *et al*, 2013; Kershaw *et al*, 2015). In agreement, these domains interact with phospholipid‐containing liposomes *in vitro* and exhibit ligand‐specific preferences for liposomes of different compositions (Suckling *et al*, 2017). Comparisons between mammalian Jagged and Delta type ligands revealed a diversity in the structures of the loops at the apex of the C2 domain which are implicated in membrane recognition in other C2 domain proteins (Suckling *et al*, 2017). A subset of missense mutations, which affect these loops in Jagged‐1, are associated with extrahepatic biliary atresia (EHBA)(Kohsaka *et al*, 2002). Purified EHBA variants show reduced Notch activation in reporter cell assays and lead to a reduction in phospholipid binding, but do not alter Notch binding (Suckling *et al*, 2017). The C2 domain may therefore have a role in tuning the activity of the Notch ligands through its lipid‐binding properties.

Mutations affecting the single Delta or Serrate (Jagged‐like) ligands in *Drosophila* have well‐characterized consequences on development (e.g. Heitzler & Simpson, 1991; Thomas *et al*, 1991; de Celis *et al*, 1996, 1997; Fleming, 1998; Bishop *et al*, 1999). Homozygous loss of ligand function leads to lethality but several defects, including wing venation abnormalities, are detected even in *Delta* heterozygotes, which have one normal gene copy (Dexter, 1914; de Celis *et al*, 1997; Huppert *et al*, 1997). As these defects occur when only one allele is mutated, it is evident that patterning is highly sensitive to ligand levels and activity. This therefore provides a powerful context in which to investigate the contributions from the apical C2 domain loops to ligand activity *in vivo*.

As the loop regions of the C2 domains are the most variable, we first set out to solve the structure of the C2 domains from the *Drosophila* Delta and Serrate ligands. This revealed similar prominent β1‐2 and β5‐6 loops to those in the C2 domain of the mammalian ligands that are thought to be responsible for the interaction with phospholipid head groups (Suckling *et al*, 2017). To test the functional contribution, we focussed on the β1‐2 loop in Delta and used CRISPR/Cas9 genome editing to delete 5 amino acids so that we could analyse the impact on Notch activity during development. *In vitro*, such *Dl^Δβ1‐2^* mutation(s) resulted in expression of a stable protein with altered phospholipid binding properties. Strikingly, *in vivo* the *Dl^Δβ1‐2^* mutation compromised ligand function, exhibiting characteristics of reduced signalling activity. Our data therefore confirm the relevance of C2 domain loops for full ligand activity and, given their ability to confer lipid binding, suggests that membrane‐binding properties are important for robust signalling.

## Results

### Structure and binding properties of the C2 domain of *Drosophila* ligands

**Figure 1:**
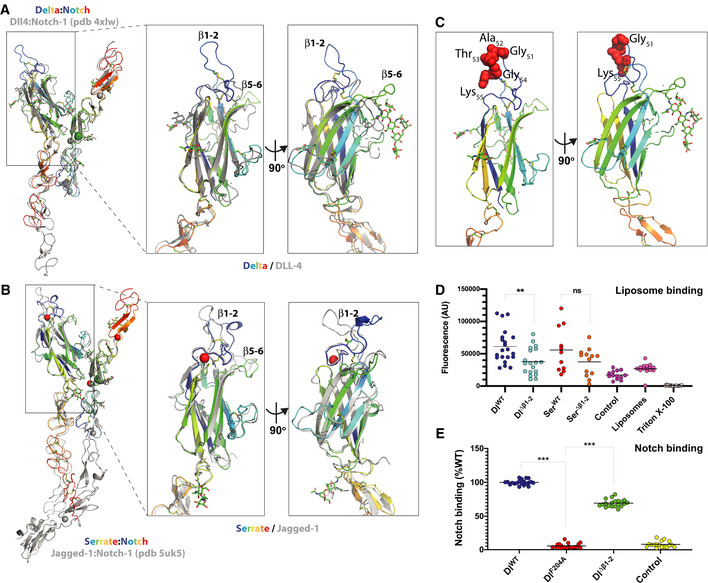
Structure and binding properties of *Drosophila* ligands A, BLeft panels. The structures of the N‐terminal regions from *Drosophila* Delta (A) and Serrate (B) are shown in a cartoon representation (rainbow coloured from blue at N terminus to red at C terminus). These have been overlaid on their mammalian equivalents DLL‐4 (A) and Jagged‐1 (B) in the context of their complexes (PDB entries 4xlw and 5uk5, respectively) with Notch‐1 (cartoon representation, coloured grey). The structure of isolated *Drosophila* Notch is also depicted in each panel (cartoon, rainbow coloured) superposed on the respective copy of mammalian Notch‐1 (cartoon, grey) for each complex. The overlays demonstrate the high degree of conservation in domain structures and arrangements between the *Drosophila* and mammalian homologues. Right panels. A close‐up view of the C2 domains of each ligand overlaid with their mammalian equivalent. These demonstrate conservation of overall fold but large differences in the apical loops, particularly in the β1‐2 and β5‐6 loops.CIsolated structure of N‐terminal Delta, with residues deleted in ∆β1‐2 highlighted as red Van Der Waals spheres.D, EBinding properties of purified *Drosophila* Delta and Serrate NE3 proteins. (D) Binding to liposomes is reduced for Delta^Δ^ *^β^* ^1‐2^, and to a more variable extent for the Serrate equivalent when using liposomes composed of PC:PS:PE‐fluoroscein (80:15:5). (E) Notch binding to *Drosophila* Delta NE3 variants. WT and Δβ1‐2 (Delta^Δ^ *^β^* ^1‐2^) both bind to Notch, unlike variant with F204A substitution in DSL domain. Comparisons were performed with a two‐tailed unpaired *t*‐test. Values are shown as scattered data points with the dark lines representing the means. ns, no significant difference, **P < 0.01; ***P < 0.0001.

**Figure EV1:**
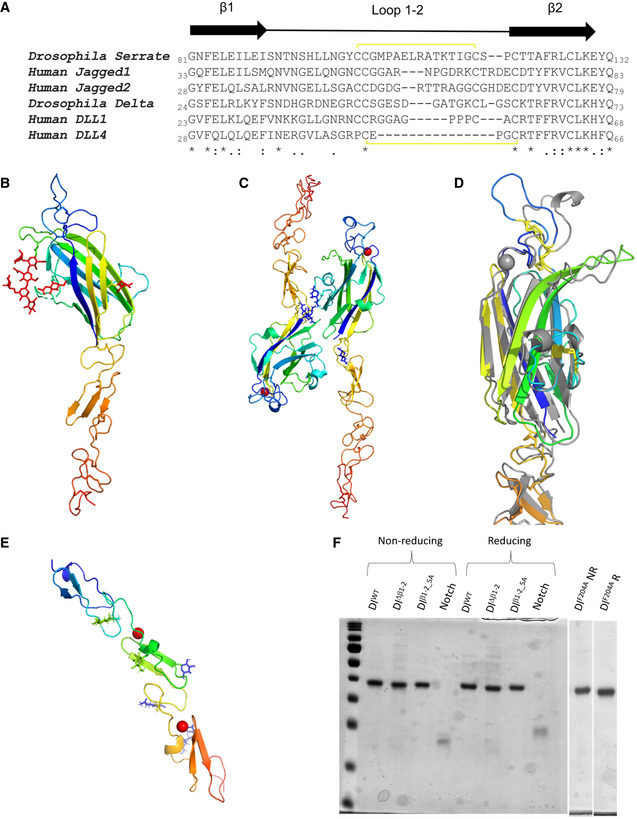
Individual structures and expression and purification of the Dl variants and Notch *in vitro* AComparison of β1‐2 loop sequences from *Drosophila* and human Notch ligands. Disulphide bond connectivity within this region is represented by the yellow brackets.B–EThe crystallographic asymmetric units are shown for the three structures (B) Delta—one copy of C2‐DSL‐EGF1 construct; (C) Serrate—two copies of C2‐DSL‐EGF1‐EGF2 construct; (D) C2 domain of *Drosophila* Delta (rainbow colouring) overlaid on the same region of *Drosophila* Serrate (grey). Disulphide bonds are shown as yellow sticks and the bound calcium ion in the Serrate structure as a grey sphere. The β1‐2 loop (blue for Delta, at top) adopts a different conformation in Delta than in Serrate despite conservation of the two stabilizing disulphides; (E) Notch—one copy of EGF11‐13. All proteins are shown in a rainbow cartoon representation coloured from blue at the N terminus of the construct to red at the C terminus, except Serrate in D (grey). In B, C, E, Glycosylation is shown in a stick representation as are the side chains to which the glycans are attached and disulphide bonds. Figure drawn using PyMol (The PyMOL Molecular Graphics System, version 2.0 Schrödinger, LLC.)FMonomeric *Drosophila* Notch ligand (N‐EGF3) and receptor (EGF11‐13) fragments were expressed in S2 cells as C‐terminal His‐tagged fusion proteins, purified by IMAC and SEC and visualized by reducing (R) and non‐reducing (NR) 10% SDS–PAGE. The Dl variants expressed for *in vitro* experiments were Dl^WT^ as control, Dl^Δ^ *^β^* ^1‐2^ where the β1‐2 loop was deleted, Dl*^β^* ^1‐2_5AA^ where the β1‐2 loop was mutated to 5 alanines. Dl^F204A^ was used as a negative control as this variant reduces Notch binding at Site 2, by altering a key residue within the ligand DSL domain. Additional weak bands in the Dl^Δ^ *^β^* ^1‐2^ sample, detected in both non‐reducing and reducing conditions, indicate carry through of slight impurities.

To determine whether the *Drosophila* ligands adopt the same arrangement as their mammalian counterparts, we solved the structures of the N‐terminal region of *Drosophila* Delta and Serrate (Fig 1) as well as the ligand‐binding region of *Drosophila* Notch (EGF11‐13; Fig EV1A–E). These were solved using molecular replacement of the individual domains from the human homologues to resolutions between 1.5 and 3.0 Å (Table 1, Fig 1). When the new *Drosophila* ligand structures were overlaid on their mammalian equivalents, Jagged‐1 and DLL‐4, it was evident that the core domain structure and arrangements of the fly ligands are highly conserved (Figs 1A–C and EV1D; RMSD 2.5 Å for Delta and 3.1 Å for Serrate) as was the structure and domain arrangement of the Notch receptor ligand‐binding region (Fig EV1D; RMSD 1.1 Å). The conserved domain arrangement allows us to model the Notch–ligand complex by overlay of the *Drosophila* structures on the earlier structures of the mammalian complexes (Luca *et al*, 2015, 2017) with this leading to no significant clashes between the Notch and ligand coordinates. Notable exceptions to the overall conserved arrangements are the β1‐2 and β5‐6 loops which exhibit different lengths and folding in the ligands. These highly variable loops protrude apically from the C2 domain core and are positioned far from the Notch‐binding interface.

**Table 1:** Data collection and refinement statistics.

	Delta C2‐DSL‐EGF1 (7ALK)	Notch EGF11‐13 (7ALJ)	Serrate C2‐DSL‐EGF1‐2 (7ALT)
Wavelength			
Resolution range	29.11–3.0 (3.107–3.0)	45.2–1.523 (1.577–1.523)	45.61–2.03 (2.103–2.03)
Space group	P 21	C 2	P 21
Unit cell	30.99 86.736 47.558 90 94.271 90	180.82 31.2858 21.7952 90 90.769 90	70.329 49.402 93.123 90 110.249 90
Total reflections	16,842 (1759)	61,424 (6192)	126,615 (12180)
Unique reflections	5,024 (520)	18,808 (1771)	38,618 (3814)
Multiplicity	3.4 (3.5)	3.3 (3.2)	3.3 (3.2)
Completeness (%)	99.84 (100.00)	98.38 (92.19)	98.89 (98.63)
Mean I/sigma(I)	4.55 (0.85)	7.75 (1.68)	11.10 (2.28)
Wilson B‐factor	33.66	20.54	31.51
R‐merge	0.2597 (1.459)	0.07143 (0.4754)	0.06213 (0.6113)
R‐meas	0.3097 (1.73)	0.08538 (0.5717)	0.0744 (0.7343)
R‐pim	0.167 (0.9225)	0.04621 (0.313)	0.04049 (0.4025)
CC1/2	0.952 (0.483)	0.996 (0.701)	0.998 (0.769)
CC*	0.988 (0.807)	0.999 (0.908)	1 (0.932)
Reflections used in refinement	5,024 (520)	18,654 (1770)	38,601 (3808)
Reflections used for R‐free	287 (33)	962 (103)	1,996 (185)
R‐work	0.2407 (0.2909)	0.2006 (0.3670)	0.2200 (0.3143)
R‐free	0.2968 (0.3939)	0.2407 (0.4334)	0.2578 (0.3285)
CC(work)	0.881 (0.588)	0.946 (0.796)	0.950 (0.787)
CC(free)	0.829 (0.310)	0.918 (0.819)	0.906 (0.770)
Number of non‐hydrogen atoms	1,902	1,024	4,255
Macromolecules	1,801	856	4,079
Ligands	99	71	58
Solvent	2	97	118
Protein residues	239	115	539
RMS(bonds)	0.003	0.017	0.005
RMS(angles)	0.58	1.40	0.81
Ramachandran favoured (%)	90.31	95.58	93.95
Ramachandran allowed (%)	9.69	4.42	5.67
Ramachandran outliers (%)	0.00	0.00	0.38
Rotamer outliers (%)	0.50	0.00	1.78
Clashscore	5.19	9.82	3.98
Average B‐factor	35.61	31.83	41.24
Macromolecules	35.01	30.95	40.91
Ligands	46.88	36.00	67.50
Solvent	19.82	36.54	39.79

Statistics for the highest‐resolution shell are shown in parentheses.

Given the structural conservation with the mammalian ligands, it is likely that the *Drosophila* proteins exhibit similar properties. Purified N‐terminal fragments (NE3 variants) were therefore used to test the liposome‐binding capability of variants in which 5 amino acids were deleted from the β1‐2 loop, hereafter referred to as Delta^Δβ1‐2^. The β1‐2 loop was selected because of its importance for phospolipid binding in other C2 domain proteins (Verdaguer *et al*, 1999; Honigmann *et al*, 2013; Hirano *et al*, 2019) and because the genomic organization (present in a single exon) meant that the equivalent mutation could be engineered *in vivo* (as described below). Using a liposome composition of phosphatidylcholine (PC): phosphatidylserine (PS): phosphatidylethanolamine‐fluoroscein (PE) (80:15:5), we could detect binding of wild‐type Delta (Delta^WT^) fragment to the liposomes as seen for mammalian Notch ligands (Fig 1D). This binding was compromised when the variable β1‐2 loop was shortened, resulting in the deletion of residues GATGK; Delta^Δβ1‐2^ fragment exhibited a significant reduction in binding when compared to that from Delta^WT^. Likewise, the equivalent fragment containing Serrate^Δβ1‐2^ loop deletion (removal of residues LRATK) also exhibited reduced binding to liposomes, although to a variable extent that was not reproducibly significant (Fig 1D). This may be due to differences in the lipid‐binding specificities because we have previously noted the heterogeneity of the C2 loop sequences in different ligand families and hypothesized that they may confer different lipid‐binding specificities (Suckling *et al*, 2017).

Purified Delta (NE3 fragment) also exhibited robust binding to a fragment of *Drosophila* Notch (dNotch EGF11‐13), which contains the core ligand‐binding sites (Fig 1E). This relies on the conventional contact sites because it is abolished by an alanine substitution in the DSL domain which replaces a key receptor‐binding residue (F204). In comparison, the variant with the loop deletion, the Delta^Δβ1‐2^ fragment, retained Notch binding as predicted from the fact that the loop is positioned far away from the Notch‐binding interface (Fig 1A–C). We attribute the small difference in Notch binding compared to the wild‐type to the slightly lower purity of the protein preparation (Fig EV1F) although we cannot rule out that the mutation causes a minor modification to the Notch interaction.

Together these data demonstrate that the C2 domain structure is conserved between species and that the properties detected in the mammalian ligands are also shared by the *Drosophila* counterparts. The main source of variability is present in the N‐terminal apical loops which nevertheless are important for liposome binding in *Drosophila* Delta as in DLL‐4 and Jagged‐1 from mammals.

### Phenotypes produced by mutations in the ligand β1‐2 loop

**Figure 2:**
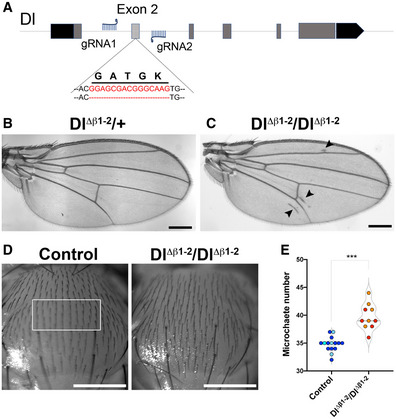
Dl β1‐2 loop mutant generated by genome editing ATwo gRNAs flanking the *Dl* Exon 2 were used to replace the exon with a modified version where 5 amino acids in the β1‐2 loop were removed. Red lettering highlights the genomic sequence of the β1‐2 loop.B, CAdult wings from *Dl^Δβ1‐2^* flies. No defects are detected in wings from *Dl^Δβ1‐2^/+* (B), Homozygous *Dl^Δβ1‐2^/Dl^Δβ1‐2^* have extra vein tissue near L5 and uneven L2 veins (arrowheads; C).DMicrochaetae are arranged in rows on the thorax of control (yw) flies; these become disordered and more dense in *Dl^Δβ1‐2^/Dl^Δβ1‐2^*. White rectangle indicates area scored for E.ENumber of microchaetes per central area (white rectangle in D) in the indicated genotypes. Data information: ***P < 0.0001 (unpaired *t*‐test). Each dot represents an individual fly, and light or dark shading indicates individuals from independent genetic crosses. On the violin plot, dashed line represents the median and the dotted lines show the quartiles. Scale bars correspond to 200 μm (B, C) and 500 μm (D).

**Figure EV2:**
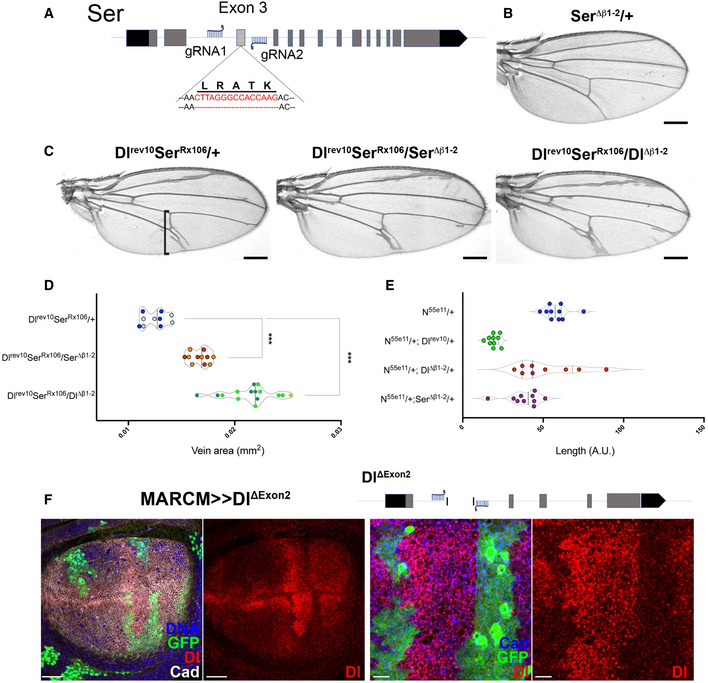
Ser β1‐2 loop mutant enhances vein phenotypes induced by reduced Dl and Ser activities Strategy to remove β1‐2 loop sequence from Exon 3 in *Serrate*. Red lettering highlights the sequence of the β1‐2 loop of Ser.Adult wings from *Ser^Δβ1‐2^* heterozygotes have no visible phenotype.Wings from *Dl^Δβ1‐2^* and *Ser^Δβ1‐2^* alleles in combination with *Dl^rev10^; Ser^Rx106^*; both mutants enhance vein thickening. Black square bracket indicates the region used for vein thickness quantification.Quantification of vein thickening in wings from females of indicated genotypes; ***P < 0.0001 (unpaired *t*‐test). Light, dark shading indicates data points from two independent replicates.Quantification of L5 vein width at intersection with the wing margin of indicated genotypes; *Dl^rev10^* strongly suppressed the small “delta” produced by *N^55e11^*/+ (*P* < 0.0001, one‐way ANOVA), combinations with *Dl^Δβ1‐2^* and *Ser^Δβ1‐2^* resulted in mild and variable suppression of borderline significance (ns and *P* < 0.05, respectively, one‐way ANOVA). On the violin plots, dashed lines represent the median and the dotted lines show the quartiles.Gene diagram showing that the two gRNAs used to replace the Dl Exon 2 also generated a novel allele where the Exon2 was removed, *Dl^ΔExon2^*. *Dl^ΔExon2^* mutant clones (green) in wing imaginal discs stained for Dl (red) and the apical marker Cadherin (blue); Dl protein is absent in the mutant cells, indicating that little or no stable protein is made most likely because the signal sequence has been deleted. Data information: Scale bars represent 200 μm (B, C), 50 μm (F) and 10 μm in the magnified image of F.

The β1‐2 and β5‐6 loops generally make important contributions to phospholipid binding in C2 domains (Verdaguer *et al*, 1999; Honigmann *et al*, 2013; Hirano *et al*, 2019). As the β1‐2 loop in the *Drosophila* ligands is encoded by a small sequence in a single exon (exon 2 of *Delta* and exon 3 of *Serrate)*, it was the most amenable to mutagenesis by genomic engineering. Therefore, in order to study the importance of this loop for Notch signalling, the endogenous exons were replaced by modified exons where the coding sequence of the loops was partially deleted by CRISPR‐mediated homologous recombination. For each of the ligands, two gRNAs were designed to flank the target exon, and the recombination of the modified exon was promoted by a complementary sequence within which the β1‐2 loop was replaced by a mutated version (*Dl^Δβ1‐2^*; Figs 2A and EV2A). Successful recombination was identified by the presence of a DsRed marker that was subsequently removed and the mutations were confirmed by sequencing of the exon. As well as generating *Dl^Δβ1‐2^* mutations, we also recovered a deletion of the entire exon 2, *Dl^ΔExon2^*, which removes a key part of the receptor‐binding region and behaves as a null allele (Fig EV2F).

Severe loss of Delta function, as with *Dl^ΔExon2^*, results in lethality. In contrast, *Dl^Δβ1‐2^* homozygotes were viable. Nevertheless, *Dl^Δβ1‐2^* adult flies exhibited several visible phenotypes. Firstly, they had ectopic wing‐vein material, with extra vein tissue detected around L2, L5 and the posterior cross‐vein (Fig 2B and C, arrowheads). Secondly, they had abnormal spacing of the microchaetae on the thorax (Fig 2D and E). Both venation and microchaetae defects are consistent with altered Notch pathway activity (Vässin & Campos‐Ortega, 1987; Heitzler & Simpson, 1991), suggesting that localized mutations affecting the β1‐2 loop impair the function of the Delta ligand.

**Figure EV3:**
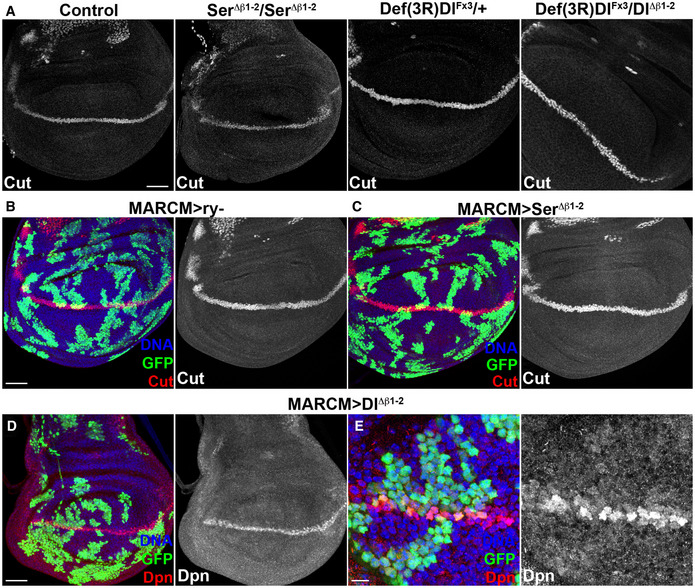
Expression of Notch targets *cut* and *dpn* is unaffected by *Dl^Δβ1‐2^* or *Ser^Δβ1‐2^* AExpression of Cut at the wing dorsal‐ventral (DV) boundary is unperturbed in wing discs of the genotypes indicated.B, CCut (red) expression is similar in wild‐type (MARCM > *ry*, green, (B) or *Ser^Δβ1‐2^* homozygous (MARCM > *Ser^Δβ1‐2^*, green, (C) clones that intersect the DV boundary. Individual Cut expression for each experiment is shown in grayscale on the right subpanels.D, E*Dl^Δβ1‐2^* homozygous clones (green) that intersect the DV boundary retain expression of Dpn (red), higher magnification in (E). Individual Dpn expression is shown in grayscale on the right subpanels. Data information: Scale bars: 50 μm (A–D) or 10 μm (E).

The defects produced by *Dl^Δβ1‐2^* were relatively mild, and there were no disruptions to the wing margin (e.g. notching). In agreement, expression of genes *cut* and *deadpan* that require high levels of Notch signalling at the d/v boundary (Micchelli *et al*, 1997; San Juan *et al*, 2012; Babaoǧlan *et al*, 2013) was not disrupted in *Dl^Δβ1‐2^* mutants (Fig EV3A) or in patches of *Dl^Δβ1‐2^* mutant cells (Fig EV3B–E). Likewise, *Ser^Δβ1‐2^* had normal wings (Fig EV2B) but exhibited mild abnormalities associated with ectopic pigmentation of joints that could also be indicative of compromised signalling. Together the data indicate that the specific deletion within the β1‐2 loop has a detectable but mild effect on Notch ligand functions.

### Ligand β1‐2 loop mutants exhibit reduced activity

**Figure 3:**
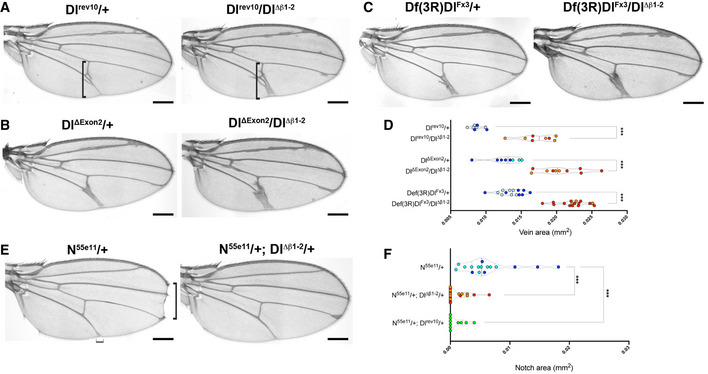
*Dl^Δβ1‐2^* enhances vein thickening from loss‐of‐function Dl alleles and suppresses *Notch* phenotype A–CRepresentative images of adult female wings in combinations of *Dl^Δβ1‐2^* with loss‐of‐function *Delta* alleles. In combinations with *Dl^rev10^* (A), *Dl^ΔExon2^* (B) or *Df(3R)Dl^Fx3^* (C), vein thickening is strongly enhanced (right panels) compared to heterozygous mutants alone (left panels). Vertical square brackets indicate the regions used for vein thickness quantification.DQuantification of wing‐vein thickness in females of the indicated genotypes.ERepresentative images of adult female wings demonstrate that *Dl^Δβ1‐2^* rescues the wing‐notching phenotype, caused by a Notch loss‐of‐function allele (*N^55e11^*). Horizontal square bracket indicates the L5 vein “delta” at the intersection with the wing margin analysed in EV2E.FQuantification of wing notching in females of the indicated genotypes, *Dl^Δβ1‐2^* rescues notching in a similar manner to *Dl^rev10^*. Data information: ***P < 0.0001 (unpaired *t*‐test). Light, dark shading indicates data points from independent genetic crosses. On the violin plots, dashed lines represent the median and the dotted lines show the quartiles. Scale bars A‐E correspond to 200 μm.

To further probe the consequences from the mutations in the C2 domain β1‐2 loop, *Dl^Δβ1‐2^* was combined in trans with previously characterized deletions (*Df(3R)Dl^Fx3^*) and loss‐of‐function (e.g. *Dl^rev10^*) *Dl* alleles. When heterozygous, the strong *Dl* alleles exhibit a robust and consistent wing‐vein phenotype, with “deltas” formed by extra vein material along several of the veins (Fig 3A–C—left panel). In combination with *Dl^Δβ1‐2^*, this phenotype was strongly enhanced, so that more of the veins were affected and they became uneven and thickened (Fig 3A–D—right panel). The enhancement of vein defects by *Dl^Δβ1‐2^* occurred in combinations with all *Dl* alleles tested. Likewise, *Ser^Δβ1‐2^* had a similar effect. Full Notch activity in the wing veins also requires Serrate, as revealed by chromosomes carrying mutations in both *Dl* and *Ser*, which have more severe phenotypes than *Dl* mutations alone despite the fact that *Ser/+* flies have normal veins (Fig EV2B). Combining *Ser^Δβ1‐2^* allele with this double‐mutant chromosome enhanced the thickening of veins in a similar manner to *Dl^Δβ1‐2^* (Fig EV2C and D). The enhanced vein phenotypes indicate that deletions within the β1‐2 loop of the C2 domain compromise ligand activity.

One unusual feature of the Notch pathway is that the ligand and receptor molecules can interact together in cis, when they are present on the same cell surface (De Celis & Bray, 1997; Micchelli *et al*, 1997). This cis‐interaction is inhibitory and may be important to set a threshold that ensures a sharp response (Sprinzak *et al*, 2010). One manifestation of this balance is that the phenotypes produced by reduced *Notch* function are suppressed when combined with a *Delta* loss‐of‐function allele (Fig 3F; De Celis & Bray, 2000). *Notch* heterozygous females have a characteristic wing‐notching phenotype (Fig 3E). When combined with *Dl^Δβ1‐2^*, the wing‐notching phenotype was suppressed to a similar extent as with a classic *Delta* allele (Fig 3E and F), suggesting that cis‐interactions are also modified in this context. *Dl^Δβ1‐2^* also gave a modest and variable modification of the vein phenotype from *Notch* heterozygotes in a similar direction (Fig EV2E). However, we note that the loop mutation is not sufficient to fully alleviate cis‐inhibition, as we did not detect ectopic target gene expression when homozygous *Dl^Δβ1‐2^* mutant clones were juxtaposed with wild‐type cells (Fig EV3D and E; (Micchelli *et al*, 1997)).

**Figure EV4:**
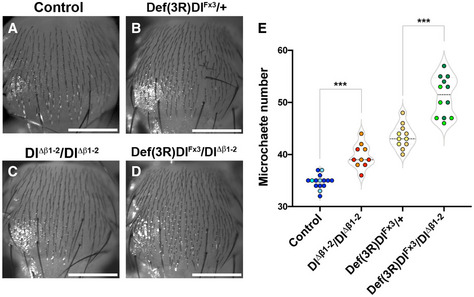
*Dl^Δβ1‐2^* compromises signalling during microchaete selection A–DMicrochaete distribution in the notum of adult flies of the following genotypes: (A) Control, *yw*; (B) *Df(3R)Dl^Fx3^/+*; (C) *Dl^Δβ1‐2^/Dl^Δβ1‐2^*; (D) *Df(3R)Dl^Fx3^/Dl^Δβ1‐2^*. Homozygous *Dl^Δβ1‐2^* mutants and *Dl^Δβ1‐2^* combined with *Df(3R)Dl^Fx3^* increases the number of the microchaetes in the notum.EQuantification of microchaete numbers which were scored in a central region (white rectangle in Fig 2D), ***P < 0.0001 (unpaired *t*‐test). Each dot represents an individual fly, and light or dark shading indicates data points from two independent crosses. On the violin plot, dashed line represents the median and the dotted lines show the quartiles. Data information: Scale bars (A–D) correspond to 500 μm.

Changes to signalling were also detected in another Notch‐dependent process, the spacing between the sensory organs, microchaetae, on the notum. In the absence of Notch signalling, an excess of sensory organ precursors are formed due to failure in lateral inhibition (Heitzler & Simpson, 1991; De Joussineau *et al*, 2003; Cohen *et al*, 2010; Sjöqvist & Andersson, 2017). Milder defects in Notch signalling lead to irregular and reduced spacing between the sensory organ precursors with the consequence that there is an increase in the number of microchaete on the adult notum as seen in flies heterozygous for a deletion of Delta (e.g. *Df(3R)Dl^Fx3^/+*; Fig EV4A,B,E). As noted above, *Dl^Δβ1‐2^* homozygous flies had an increased density of microchaetae compared to wild‐type (Figs 2D and E and EV4C and E) and in combination with strong Delta alleles, *Dl^Δβ1‐2^* led to a further increase in microchaetae numbers (Fig EV4D and E; *Df(3R)Dl^Fx3^/Dl^Δβ1‐2^*). Thus, as with the vein formation, the defects in microchaetae spacing indicate a reduced signalling potential for ligands with a shortened β1‐2 loop, despite the fact that this change should not disrupt binding to the receptor per se (see Fig 1E).

### *Dl^Δβ1‐2^* has compromised Notch signalling in photoreceptor fate decisions

**Figure 4:**
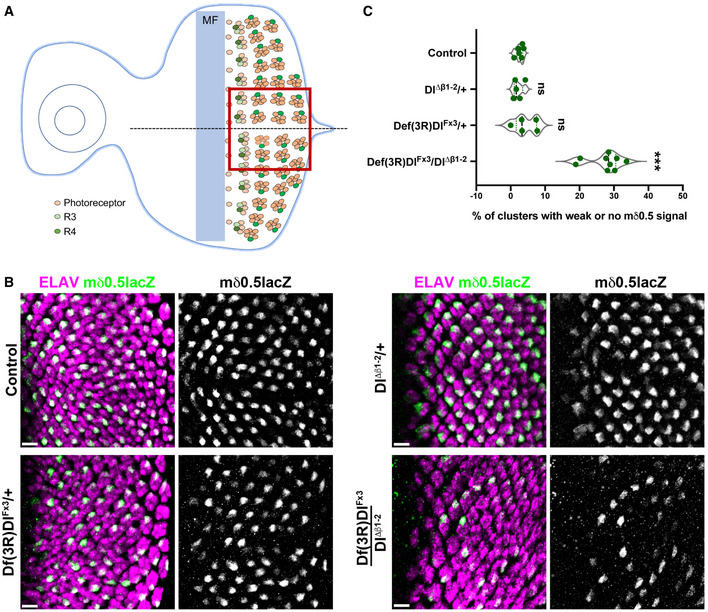
*Dl^Δβ1‐2^* has compromised Notch response in photoreceptor fate decisions Schematic representation of Notch reporter *E(spl)mδ0.5* expression during photoreceptor differentiation. Expression is initiated in R3 and R4 of the 5‐cell pre‐cluster and becomes restricted to R4 as Notch activity resolves. Light orange indicates photoreceptors with R3 in light green and R4 in dark green. MF marks the morphogenetic furrow, boxed region indicates the region shown in B.Equatorial region of eye imaginal discs where *E(spl)mδ0.5* expression (green) becomes restricted to a single photoreceptor in each cluster (magenta), as detected in control and *Dl^Δβ1‐2^*/+ discs (top panels). In *Df(3R)Dl^Fx3^* /+ and *Dl^Δβ1‐2^/Df(3R)Dl^Fx3^* discs (bottom panels), E(spl)mδ0.5 expression is reduced (*Df(3R)Dl^Fx3^* /+) or absent from several clusters (*Dl^Δβ1‐2^/Df(3R)Dl^Fx3^*) indicative of reduced Notch signalling. Scale bars correspond to 10 μm.Proportion of photoreceptors clusters that fail to express the *E(spl)mδ0.5* reporter in the indicated genotypes. ns, no significant difference, ***P < 0.0001 (one‐way ANOVA). On the violin plot, dashed line represents the median and the dotted lines show the quartiles.

**Figure EV5:**
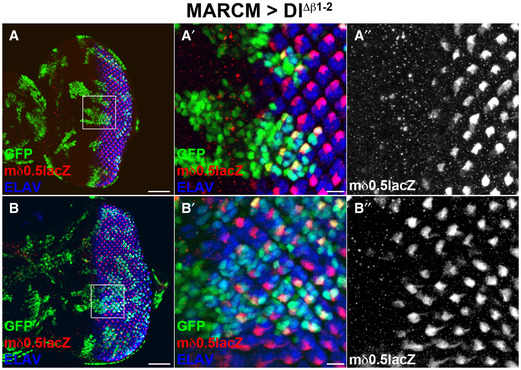
*E(spl)mδ0.5* expression is retained in homozygous *Dl^Δβ1‐2^* clones *Dl^Δβ1‐2^* homozygous clones (green) in eye imaginal discs with *E(spl)mδ0.5* (red) reporter expression, ELAV (blue) marks all photoreceptors. (A’A”) Higher magnification of the equatorial region (marked by white square in A), *E(spl)mδ0.5* (red, A’; white, A”) is detected in R4 of wild‐type and mutant clusters, including clusters where R3 is mutant.Large homozygous *Dl^Δβ1‐2^* clone (green), *E(spl)mδ0.5* (red) expression is unaffected, ELAV (blue) marks all photoreceptors. (B’, B”) Higher magnification of the equatorial region (marked by white square in B), *E(spl)mδ0.5* (red, B’; white, B”) is detected in R4 of wild‐type and mutant clusters, including clusters where R3 is mutant. Data information: Scale bars correspond to 50 μm (A, B) or 10 μm (A’, B’).

Flies homozygous for *Dl^Δβ1‐2^* also had mild roughening of the eyes. Notch activity is required at several stages in the development of the photoreceptors, including in the specification of R4 and R7 photoreceptors. The sequential differentiation of the eight neuronal photoreceptors (R cells) is initiated when a wave of differentiation (called morphogenetic furrow or MF) spreads from the posterior to the anterior region of the eye imaginal disc (Şahin & Çelik, 2013; Fig 4A). Notch activity in one cell of the five‐cell cluster specifies R4 cell fate and can be detected by the expression of *E(spl)mδ0.5‐lacZ*, containing the Notch responsive *E(spl)mδ* enhancer ((Cooper & Bray, 1999); Fig 4 A and B). Reducing the levels of Delta, as seen in Delta heterozygotes *Df(3R)Dl^Fx3^*/+, led to more variable expression of *E(spl)mδ0.5* (Fig 4B). This was further enhanced in combination with *Dl^Δβ1‐2^*, so that many of the ommatidia exhibited very low levels of expression (Fig 4 B and C). No similar reduction occurred with *Dl^Δβ1‐2^* heterozygotes (Fig 4B) nor clones of *Dl^Δβ1‐2^* homozygous mutant cells (Fig EV5A and B’’) arguing that the decrease in activity in these conditions is not below the threshold needed for *E(spl)mδ0.5* activation. Nevertheless, the fact that the *Dl^Δβ1‐2^* enhances the phenotype from the Delta deletion is consistent with it being compromised for productive Notch signalling.

### C2 Domain β1‐2 loop mutation does not impair Delta trafficking

**Figure 5:**
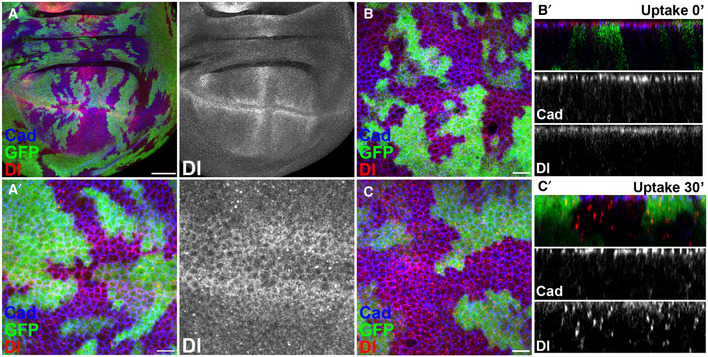
*Dl^Δβ1‐2^* exhibits normal sub‐cellular localization Apical view of wing imaginal disc with homozygous *Dl^Δβ1‐2^* clones (GFP negative) stained for Dl (red) and Cadherin (blue). (A’) Z‐projection of apical layers spanning a *Dl^Δβ1‐2^* clone (GFP negative) located at the DV boundary. No change in apical localization of Dl (red) and Cadherin (blue) is detected. Panels on the right show the Dl (grayscale) apical localization on the wing imaginal disc.Uptake assay at *t* = 0. After exposure to extracellular anti‐Dl antibody, Dl protein (red) is detected at similar levels apical to Cadherin (blue) in wild‐type (GFP) and *Dl^Δβ1‐2^* mutant tissue (GFP negative). (B’) Cross‐sectional view of B, Dl protein (red) is present apically relative to Cadherin (Blue) which marks adherens junctions. Middle and lower panels show the cross section in grayscale of the apical marker Cadherin (Cad) and Delta (Dl), respectively.Uptake assay after 30 min, internalized anti‐Dl (red) enters the endocytic route and in the cross‐sectional view (C’) can similarly be detected as puncta along the cell axis in wild‐type (GFP) and homozygous *Dl^Δβ1‐2^* (GFP negative) tissue. Middle and lower panels show the cross section in grayscale of the apical marker Cadherin (Cad) and Delta (Dl), respectively. Data information: Scale bars: A, 50 μm; A’ B and C, 10 μm.

Our results indicate that the β1‐2 loop region of Delta C2 domain is required for full functionality. To investigate whether this involves a change in the localization or trafficking of Delta, we generated mutant clones in the wing disc, a tissue where the expression and localization of the ligand is well characterized. In late third instar stages, the expression of Delta is particularly enriched in two stripes flanking the DV boundary and in longitudinal stripes that prefigure the prospective wing veins (Fig 5A and A’). In all regions of the disc, Dl^Δ^ *^β^* ^1‐2^ exhibited normal expression levels and it appeared to be localized at the apical membranes, at similar levels to wild‐type Delta.

To confirm that the mutant protein was present on the cell surface, we performed an antibody uptake assay (Le Borgne & Schweisguth, 2003). Wing imaginal discs were incubated *ex vivo* with an anti‐Dl antibody recognizing the extracellular domain at 4°C. Excess antibody was then washed away, and the tissues transferred to a permissive temperature (25°C) for 0 or 30 min so that the membrane localization, uptake and trafficking of bound antibody could be measured (Gomez‐Lamarca *et al*, 2015). At zero minutes when antibody was bound to Delta on the cell surface, similar levels were detected in control regions and in *Dl^Δβ1‐2^* mutant clones (Fig 5B and B’), indicating that the mutant protein was present on the cell surface. When endocytosis was allowed to proceed for 30 min, antibody‐bound Delta accumulated in puncta throughout the epithelial cells in both wild‐type and Dl^Δ^ *^β^* ^1‐2^ tissue (Fig 5C and C’). The uptake assays confirm therefore that the mutated protein reaches the cell surface normally and that its trafficking following endocytic uptake is not grossly affected, although we cannot rule out a subtle change.

## Discussion

C2 domain phospholipid binding properties are essential for membrane targeting of many intracellular proteins. Notch ligands are unusual in having an extracellular N‐terminal C2 domain (Chillakuri *et al*, 2013; Kershaw *et al*, 2015). This structure is present in all the human Notch ligands and retains the capacity to interact with liposomes (Suckling *et al*, 2017). Here, we showed that *Drosophila* Delta and Serrate also contain a globular C2 domain that confers the ability to bind to phospholipid‐containing liposomes *in vitro*. The C2 domain structures are highly conserved, differing only in the length and orientation of several loops. A deletion mutation affecting one of these, a loop between the β1 and β2 strands of the C2 domain core, was sufficient to compromise liposome binding. This loop might therefore help to generate a “pocket” capable of interacting with a specific type of lipid, for example phospholipid/glycosphingolipid and in this way influence productive Notch signalling.

**Figure 6:**
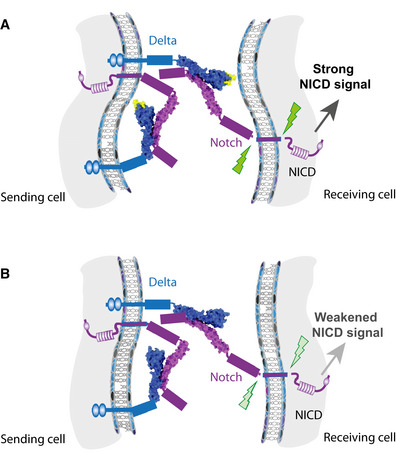
Schematic summarizing roles for ligand β1‐2 loop A, BThe C2, DSL domains and N‐terminal EGF repeat in Delta (cyan) and the ligand‐binding region (EGF11‐13) of Notch (magenta) are renditions from the structures obtained (Fig 1), other regions of the molecules are represented not to scale. The amino acids in the β1‐2 loop of Delta are highlighted in yellow (A). (A) The interaction of Delta (cyan) in *trans* with the Notch receptor (magenta) is augmented by the C2 domain, possibly through contacts of the β1‐2 loop (yellow), with the “receiving” cell membrane, to yield highest levels of signalling (black arrow; green indicates ligand induced cleavages). Phospholipid contacts from β1‐2 loop in the same cell could also influence cis‐interactions between Delta and Notch in the same cell. (B) A deletion of 5 amino acids within C2 domain β1‐2 loop (no yellow) disrupts phospholipid interactions but does not prevent Delta from interacting with Notch. Activation of Notch signalling is weakened (grey arrow) and phenotypes from transheterozygous combinations suggest that cis‐interactions between Delta and Notch are also modulated.

A subset of human *Jagged‐1* mutations that affect the loops at the apex of the C2 domain are associated with extrahepatic biliary atresia suggesting these regions are important for tuning the Notch signal in physiological contexts (Kohsaka *et al*, 2002; Suckling *et al*, 2017). Our results, from CRISPR engineering β1‐2 loop mutations in *Drosophila* Delta and Serrate, support the conserved functional importance of the C2 domain loops. The mutated Delta exhibited reduced signalling activity in several different developmental contexts. The compromised signalling was most evident in genetic combinations with a strong loss‐of‐function allele or deletion of the locus and was manifest by enhanced vein thickening, extra sensory bristles and reduced signalling during photoreceptor fate choice, although there were no overt effects at the dorsal‐ventral boundary. All of the processes affected involve highly dynamic signalling and are sensitive to subtle changes in signalling as evident from the defects in animals with reduced dosage of wild‐type Delta (*Df(3R)Dl^Fx3^*/+). These results are consistent with the model that C2 domain loop regions are important for fine‐tuning the Notch signal (Fig 6A and B), as suggested by *in vitro* results, where the binding of fluorescent liposomes to Jagged was modified/enhanced in the presence of a Notch‐1 11‐13 fragment, suggesting a coupling between C2 domain lipid binding and Notch binding, and by the changes in Notch activation seen with EHBA and related loop variants (Suckling *et al*, 2017).

There are several models for how C2 domain‐mediated membrane interactions might impact on signalling. One possibility is that the spatial or temporal residence of Delta in the membrane may be affected by the C2 domain interactions. Evidence suggests that relative pools of the receptor and ligands, rather than absolute concentrations, are important for refining signalling outcomes due to the balance between cis‐inhibition and trans‐activation (Sprinzak *et al*, 2010). Models based on this relationship inferred that intrinsic noise would cause the width of the vein to become irregular, one characteristic of the phenotype produced from *Dl^Δβ1‐2^*. Loss of interaction with certain types of lipids might bias how the ligand interacts with the receptor. For example, it could shift in favour of inhibition, producing a generalized reduction in signalling, despite there being similar amounts of proteins on the cell surface. However, the ability of *Dl^Δβ1‐2^* to suppress the phenotype from reduced Notch at the wing margin argues that cis‐inhibition is also compromised by the loop mutation in some contexts. This makes it more likely that C2 domain interactions fine‐tune both activating and inhibitory interactions, perhaps by modulating the length of time the ligand is diffusing in the membrane (Khait *et al*, 2016), and that the precise consequences may differ depending on the relative amounts of ligand and receptor present.

In summary, our structure‐guided approach to make defined changes in the endogenous ligands has demonstrated the *in vivo* relevance of C2 domain loops for full activity in the physiological setting (Fig 6A and B). This approach has uncovered subtle functional requirements that would unlikely be detected using *in vitro* or *in vivo* methods alone, highlighting the importance of using interdisciplinary methods to fully elucidate function.

## Materials and Methods

### Protein expression, crystallization and structure determination

Codon optimized open reading frames for constructs (synthesized by GeneArt®, Life Technologies Ltd., Paisley, UK), with recommended BiP signal peptide (for secretion), were subcloned into expression vector pEXS2‐2 (Expres2ion® Biotechnologies, Horsholm, Denmark) using *Eco*RI and *Not*I restriction sites (see Table EV1 for primer sequences). Each construct was expressed as a monomer with a C‐terminal 8xHis tag to facilitate purification. Purification was as described in Suckling *et al* (2017). *Drosophila* (d) Delta and Serrate NE3 (C2 domain‐DSL, EGF1, EGF2, EGF3) constructs were used for liposome‐ and Notch‐binding assays. NE3 (residues 1–332 Delta; 1–388 Serrate), NE2 (residues 1–293 Delta, residues 1–349 Serrate), NE1 (1–259 Delta, 1–314 Serrate) constructs were used to set up preliminary crystal trials. Delta NE1 and Serrate NE2 constructs produced best diffracting crystals. NotchEGF11‐13 was produced using the same expression system, and the purified cleaved form used for crystallization.

The Notch receptor construct was concentrated to 24.2 mg/ml in a buffer A (10 mM Tris pH 7.5, 150 mM NaCl, 10 mM CaCl_2_) and crystallized by the sitting drop method from 200 nl + 200 nl drops with mother liquor 0.1 M MES pH 6.5, 1.8 M ammonium sulphate, 0.01 M cobalt chloride. Crystals were cryoprotected by addition of 25% ethylene glycol and data collected at the European Synchrotron Radiation Facility, beamline ID29. Serrate was concentrated to 15.8 mg/ml in buffer A and crystallized by the sitting drop method from 200 nl + 200 nl drops with mother liquor 0.1 M imidazole malate pH 7, 25% PEG4K and cryoprotected by addition of 25% ethylene glycol, 20 mM CaCl_2_. Data were collected at Diamond Light Source, beamline I02. Delta was concentrated to 17.7 mg/ml and crystallized by the sitting drop method from 200 nl + 200 nl drops with mother liquor 0.1 M Tris pH 8.5, 0.2 M MgCl_2_, 30% PEG4K and cryoprotected with 25% glycerol, 20 mM CaCl_2_. Data were collected at Diamond Light Source, beamline I04.

All structures were solved by molecular replacement using separated domains from the human homologues using program PHASER (McCoy *et al*, 2007) from program suite CCP4 (The CCP^4^ suite: programs for protein crystallography, 1994) rebuilt using BUCCANEER (Cowtan, 2006) and COOT (Emsley *et al*, 2010) and refined in PHENIX (Liebschner *et al*, 2019). For all constructs, data processing and model statistics are described in Table 1. Coordinates and data are deposited in the Protein Data Bank with accession codes 7ALJ, 7ALT, 7ALK for Notch, Serrate and Delta, respectively.

### Liposome‐ and Notch‐binding assays

Liposome‐binding assays were carried out as described in Suckling *et al* (2017) using purified Delta/Serrate variants and liposomes comprising phosphatidylcholine (PC): phosphatidylserine (PS): phosphatidylethanolamine‐fluoroscein (PE) in a 80:15:5 ratio. Liposomes were prepared as described in Chillakuri *et al* (2013). Notch‐binding assays were carried out as described in Suckling *et al* (2017) using purified Delta variants and Notch EGF 11‐13. The negative control Delta F204A variant reduces Notch/ligand binding at Site 2, by altering a key residue within the ligand DSL domain Notch‐binding loop. We note there is some slight variability in the purity of the protein preparations (see Fig EV1F).

### *Drosophila melanogaster* strains and genetics

All *Drosophila melanogaster* stocks were grown on standard medium at 25°C. Alleles are as described in Flybase (Thurmond *et al*, 2019) and in particular the following were used to sensitize the genetic background: *Dl^rev10^* (Heitzler & Simpson, 1991), *Dl^rev10^, Ser^Rx106^* (Thomas *et al*, 1991), *Df(3R)Dl^Fx3^* (Vässin & Campos‐Ortega, 1987), *N^55e11^* (#BL28813). *Dl^Δβ1‐2^* clones were generated using FRT‐mediated recombination (Xu & Rubin, 1993) – recombination was promoted by heat shock of 1 h at 37°C 72 h prior to dissection and analysis. The *E(spl)mδ0.5* reporter was used for analysis of R3/R4 determination in eye imaginal discs, (Cooper & Bray, 1999).

### Generation of β1‐2 loop Notch ligands mutants using CRISPR/Cas9

Lines were generated by CRISPR‐mediated homology repair (HR) strategy. As described in Figs 3 and EV2, two guideRNAs were designed to flank the target exon coding the β1‐2 loop (see Table EV1 for primer sequences) and cloned into the guide RNA expression pCFD4 vector (Addgene #49411). The exon of interest and homology arms were cloned into donor template plasmid pHD‐ScarlessDsRed (Addgene # 64703) using the Gibson Assembly Protocol (see Table EV1 for primer sequences). Modifications to the exons were made using standard mutagenesis and PCR amplification prior to the co‐injection of the guide RNAs and the donor template constructs into *nos‐Cas9* (#BL54591) embryos. Modifications included the following: 1) deletion of 15 bp within β1‐2 loop of Dl (*Dl^Δβ1‐2^*); 2) deletion of 15 bp within β1‐2 loop from *Ser* Exon 3 (*Ser^Δβ1‐2^*); 3) deletion of region between the two gRNAs (*Dl^ΔExon2^*). ∆Exon 2 could in principle produce a modified protein, the deletion would be in frame, but it would lack any signal peptide. As no residual protein was detected by antibody staining (Fig EV2F), the product is either not made or is unstable. Engineered flies were identified by expression of DsRed in the eyes and verified by genomic PCR sequencing. The transposable element containing the DsRed was removed subsequently by crossing to flies carrying PiggyBac Transposase (#BL32070).

### Immunostainings

The following primary antibodies were used for immunofluorescence staining: Goat anti‐GFP (1:200, Abcam, ab6673), Mouse anti‐Cut (1:20, Developmental studies hybridoma bank (DSHB)), Rat anti‐DE‐Cad2 (1:200, DSHB), Mouse anti‐Delta (1:30, DSHB), Guinea pig anti‐Delta (1:2,000, a gift from Mark Muskavitch, (Huppert *et al*, 1997)), Guinea pig anti‐Dpn (1:2,000, a gift from Christos Delidakis), Mouse anti‐NECD (1:50, DSHB), Rat anti‐ELAV (1:200, DSHB), Mouse anti‐β‐Gal (1:1,000, Promega, Z378A). Uptake assay was performed as described previously (Gomez‐Lamarca *et al*, 2015).

### Adult tissues analysis

For the analysis of the adult fly wings, female flies were collected in 70% ethanol for 2 h, rehydrated in PBS and one wing per fly was isolated and mounted in a 50% glycerol solution. To analyse the microchaete number, flies were collected in 70% ethanol for 2 h, rehydrated in PBS 1× and mounted on apple juice agar plates for imaging.

### Imaging and statistical analysis

Immunostaining samples were imaged with Leica TCS SP8 microscopes (CAIC, University of Cambridge) at 40× magnification and 512/512 or 1,024/1,024 pixel resolutions. Images of the adult wings were taken using a Zeiss Axiophot microscope, and images of the adult Notum were taken using the Leica MZ10F coupled with a camera Leica DFC3000G. ImageJ software was used to analyse images and polygon tool was used to measure the vein area on the region limited by the CV2, L4 and L5 veins on adult wings. The measurement of the wing notching was done by determining the tissue missing with the polygon tool after superimposing wings of the described genotypes with the reference wild‐type wing. The number of microchaete was assayed using a fixed area as reference on the Notum, as depicted by the white box on Fig 2D. For the analysis of the Dl and Notch trafficking in *Dl^Δβ1‐2^* mutant clones, a projection of 3‐cell diameter was performed after re‐slicing the images into the XZY axis in ImageJ software.

Statistics were calculated with GraphPad Prism. Comparisons between two groups were performed with a two‐tailed unpaired *t*‐test. Statistical differences among various groups were assessed with ordinary one‐way ANOVA by comparison to the mean of the control column. In the figures and figure legends, ns indicates no significant difference; *P < 0.1; **P < 0.001; ***P < 0.0001.

## Author contributions

TM and SJB designed the *in vivo* experiments. TM performed the *in vivo* experiments. TM and SJB analysed the data. PAH and SML designed the *in vitro* experiments. BK, YM and RS purified ligand and receptor proteins. RS and SJ performed structure determination. YM performed the Notch‐binding experiments. BK performed liposome‐binding assays. BK, YM, RS, PAH and SML analysed data. TM, PAH, SML and SJB wrote the manuscript.

## Conflict of interest

The authors declare that they have no conflict of interest.

## Supporting information



Expanded View Figures PDF

Table EV1

## Data Availability

Coordinates and data have been deposited in the RCSB Protein Data Bank (https://www.rcsb.org/) with accession codes 7ALJ, 7ALT, 7ALK for Notch, Serrate and Delta, respectively.

## References

[embr202152729-bib-0001] Babaoǧlan AB, Housden BE, Furriols M, Bray SJ (2013) Deadpan contributes to the robustness of the notch response. PLoS One 8: e75632 24086596 10.1371/journal.pone.0075632 PMC3782438

[embr202152729-bib-0002] Bishop SA, Klein T, Arias AM, Couso JP (1999) Composite signalling from Serrate and Delta establishes leg segments in *Drosophila* through Notch. Development 126: 2993–3003 10357942 10.1242/dev.126.13.2993

[embr202152729-bib-0003] Boukhatmi H, Martins T, Pillidge Z, Kamenova T, Bray S (2020) Notch mediates inter‐tissue communication to promote tumorigenesis. Curr Biol 30: 1809–1820 32275875 10.1016/j.cub.2020.02.088

[embr202152729-bib-0004] Bray SJ (2006) Notch signalling: a simple pathway becomes complex. Nat Rev Mol Cell Biol 7: 678–689 16921404 10.1038/nrm2009

[embr202152729-bib-0005] Bray SJ (2016) Notch signalling in context. Nat Rev Mol Cell Biol 17: 722–735 27507209 10.1038/nrm.2016.94

[embr202152729-bib-0006] de Celis JF, Garcia‐Bellido A, Bray SJ (1996) Activation and function of Notch at the dorsal‐ventral boundary of the wing imaginal disc. Development 122: 359–369 8565848 10.1242/dev.122.1.359

[embr202152729-bib-0007] Chillakuri CR, Sheppard D, Ilagan MXG, Holt LR, Abbott F, Liang S, Kopan R, Handford PA, Lea SM (2013) Structural analysis uncovers lipid‐binding properties of notch ligands. Cell Rep 5: 861–867 24239355 10.1016/j.celrep.2013.10.029 PMC3888931

[embr202152729-bib-0008] Cohen M, Georgiou M, Stevenson NL, Miodownik M, Baum B (2010) Dynamic filopodia transmit intermittent Delta‐Notch signaling to drive pattern refinement during lateral inhibition. Dev Cell 19: 78–89 20643352 10.1016/j.devcel.2010.06.006

[embr202152729-bib-0009] Collaborative Computational Project, Number 4 (1994) The CCP4 suite: programs for protein crystallography. Acta Crystallogr D Biol Crystallogr 50: 760–763 15299374 10.1107/S0907444994003112

[embr202152729-bib-0010] Cooper MTD, Bray SJ (1999) Frizzled regulation of Notch signalling polarizes cell fate in the *Drosophila* eye. Nature 397: 526–530 10028969 10.1038/17395

[embr202152729-bib-0011] Cordle J, Johnson S, Zi Yan Tay J, Roversi P, Wilkin MB, de Madrid BH, Shimizu H, Jensen S, Whiteman P, Jin B*et al* (2008) A conserved face of the Jagged/Serrate DSL domain is involved in Notch trans‐activation and cis‐inhibition. Nat Struct Mol Biol 15: 849–857 18660822 10.1038/nsmb.1457 PMC2669539

[embr202152729-bib-0012] Cowtan K (2006) The Buccaneer software for automated model building. 1. Tracing protein chains. Acta Crystallogr D Biol Crystallogr 62: 1002–1011 16929101 10.1107/S0907444906022116

[embr202152729-bib-0013] D’Souza B, Miyamoto A, Weinmaster G (2008) The many facets of Notch ligands. Oncogene 27: 5148–5167 18758484 10.1038/onc.2008.229 PMC2791526

[embr202152729-bib-0014] de Celis JF, Bray S, Garcia‐Bellido A (1997) Notch signalling regulates veinlet expression and establishes boundaries between veins and interveins in the *Drosophila* wing. Development 124 **:** 1919–1928 9169839 10.1242/dev.124.10.1919

[embr202152729-bib-0015] De Celis JF, Bray S (1997) Feed‐back mechanisms affecting Notch activation at the dorsoventral boundary in the *Drosophila* wing. Development 124: 3241–3251 9310319 10.1242/dev.124.17.3241

[embr202152729-bib-0016] De Celis JF, Bray SJ (2000) The Abruptex domain of Notch regulates negative interactions between Notch, its ligands and Fringe. Development 127: 1291–1302 10683181 10.1242/dev.127.6.1291

[embr202152729-bib-0017] De Joussineau C, Soulé J, Martin M, Anguille C, Montcourrier P, Alexandre D (2003) Delta‐promoted filopodia mediate long‐range lateral inhibition in *Drosophila* . Nature 426: 555–559 14654840 10.1038/nature02157

[embr202152729-bib-0018] Dexter JS (1914) The analysis of a case of continuous variation in *Drosophila* by a study of its linkage relations. Am Nat 48: 712–758

[embr202152729-bib-0019] Emsley P, Lohkamp B, Scott WG, Cowtan K (2010) Features and development of Coot. Acta Crystallogr D Biol Crystallogr 66: 486–501 20383002 10.1107/S0907444910007493 PMC2852313

[embr202152729-bib-0020] Fleming RJ (1998) Structural conservation of Notch receptors and ligands. Semin Cell Dev Biol 9: 599–607 9918871 10.1006/scdb.1998.0260

[embr202152729-bib-0021] Gomez‐Lamarca MJ, Snowdon LA, Seib E, Klein T, Bray SJ (2015) Rme‐8 depletion perturbs Notch recycling and predisposes to pathogenic signaling. J Cell Biol 210: 303–318 26169355 10.1083/jcb.201411001 PMC4508892

[embr202152729-bib-0022] Heitzler P, Simpson P (1991) The choice of cell fate in the epidermis of *Drosophila* . Cell 64: 1083–1092 2004417 10.1016/0092-8674(91)90263-x

[embr202152729-bib-0023] Hirano Y, Gao Y‐G, Stephenson DJ, Vu NT, Malinina L, Simanshu DK, Chalfant CE, Patel DJ, Brown RE (2019) Structural basis of phosphatidylcholine recognition by the C2–domain of cytosolic phospholipase A^2^α. Elife 8: e44760 31050338 10.7554/eLife.44760 PMC6550875

[embr202152729-bib-0024] Honigmann A, van den Bogaart G, Iraheta E, Risselada HJ, Milovanovic D, Mueller V, Müllar S, Diederichsen U, Fasshauer D, Grubmüller H*et al* (2013) Phosphatidylinositol 4,5‐bisphosphate clusters act as molecular beacons for vesicle recruitment. Nat Struct Mol Biol 20: 679–686 23665582 10.1038/nsmb.2570 PMC3676452

[embr202152729-bib-0025] Huang H, Kornberg TB (2015) Myoblast cytonemes mediate Wg signaling from the wing imaginal disc and Delta‐Notch signaling to the air sac primordium. Elife 4: 1–22 10.7554/eLife.06114 PMC4423120 25951303

[embr202152729-bib-0026] Hunter GL, He L, Perrimon N, Charras G, Giniger E, Baum B (2019) A role for actomyosin contractility in Notch signaling. BMC Biol 17: 1–15 30744634 10.1186/s12915-019-0625-9 PMC6369551

[embr202152729-bib-0027] Huppert SS, Jacobsen TL, Muskavitch MAT (1997) Feedback regulation is central to Delta‐Notch signalling required for *Drosophila* wing vein morphogenesis. Development 124: 3283–3291 9310323 10.1242/dev.124.17.3283

[embr202152729-bib-0028] Kershaw NJ, Church NL, Griffin MDW, Luo CS, Adams TE, Burgess AW (2015) Notch ligand delta‐like1: X‐ray crystal structure and binding affinity. Biochem J 468: 159–166 25715738 10.1042/BJ20150010

[embr202152729-bib-0029] Khait I, Orsher Y, Golan O, Binshtok U, Gordon‐Bar N, Amir‐Zilberstein L, Sprinzak D (2016) Quantitative analysis of delta‐like 1 membrane dynamics elucidates the role of contact geometry on notch signaling. Cell Rep 14: 225–233 26748704 10.1016/j.celrep.2015.12.040

[embr202152729-bib-0030] Kohsaka T, Yuan Z, Guo S, Tagawa M, Nakamura A, Nakano M, Kawasasaki H, Inomata Y, Tanaka K, Miyauchi J (2002) The significance of human jagged 1 mutations detected in severe cases of extrahepatic biliary atresia. Hepatology 36: 904–912 12297837 10.1053/jhep.2002.35820

[embr202152729-bib-0031] Kopan R, Ilagan MXG (2009) The canonical Notch signaling pathway: unfolding the activation mechanism. Cell 137: 216–233 19379690 10.1016/j.cell.2009.03.045 PMC2827930

[embr202152729-bib-0032] Kovall RA (2008) More complicated than it looks: assembly of Notch pathway transcription complexes. Oncogene 27: 5099–5109 18758478 10.1038/onc.2008.223

[embr202152729-bib-0033] Kovall RA, Blacklow SC (2010) Mechanistic insights into Notch receptor signaling from structural and biochemical studies. Curr Top Dev Biol 92: 31–71 20816392 10.1016/S0070-2153(10)92002-4

[embr202152729-bib-0034] Kovall RA, Gebelein B, Sprinzak D, Kopan R (2017) The canonical notch signaling pathway: structural and biochemical insights into shape, sugar, and force. Dev Cell 41: 228–241 28486129 10.1016/j.devcel.2017.04.001 PMC5492985

[embr202152729-bib-0035] Le Borgne R, Schweisguth F (2003) Unequal segregation of neuralized biases Notch activation during asymmetric cell division. Dev Cell 5: 139–148 12852858 10.1016/s1534-5807(03)00187-4

[embr202152729-bib-0036] Liebschner D, Afonine PV, Baker ML, Bunkóczi G, Chen VB, Croll TI, Hintze B, Hung LW, Jain S, McCoy AJ*et al* (2019) Macromolecular structure determination using X‐rays, neutrons and electrons: recent developments in Phenix. *Acta Crystallogr. Sect. D* . Struct Biol 75: 861–877 10.1107/S2059798319011471 PMC6778852 31588918

[embr202152729-bib-0037] Luca VC, Jude KM, Pierce NW, Nachury MV, Fischer S, Garcia KC (2015) Structural basis for Notch1 engagement of Delta‐like 4. Science 347: 847–853 25700513 10.1126/science.1261093 PMC4445638

[embr202152729-bib-0038] Luca VC, Kim BC, Ge C, Kakuda S, Wu D, Roein‐Peikar M, Haltiwanger RS, Zhu C, Ha T, Garcia KC (2017) Notch‐Jagged complex structure implicates a catch bond in tuning ligand sensitivity. Science 355: 1320–1324 28254785 10.1126/science.aaf9739 PMC5459593

[embr202152729-bib-0039] Mašek J, Andersson ER (2017) The developmental biology of genetic Notch disorders. Development 144: 1743–1763 28512196 10.1242/dev.148007

[embr202152729-bib-0040] McCoy AJ, Grosse‐Kunstleve RW, Adams PD, Winn MD, Storoni LC, Read RJ (2007) Phaser crystallographic software. J Appl Crystallogr 40: 658–674 19461840 10.1107/S0021889807021206 PMC2483472

[embr202152729-bib-0041] Micchelli CA, Rulifson EJ, Blair SS (1997) The function and regulation of cut expression on the wing margin of *Drosophila*: Notch, Wingless and a dominant negative role for Delta and Serrate. Development 124: 1485–1495 9108365 10.1242/dev.124.8.1485

[embr202152729-bib-0042] Monticone G, Miele L (2021) Notch pathway: a journey from notching phenotypes to cancer immunotherapy. Adv Exp Med Biol 1287: 201–222 33034034 10.1007/978-3-030-55031-8_13

[embr202152729-bib-0043] Nowell CS, Radtke F (2017) Notch as a tumour suppressor. Nat Rev Cancer 17: 145–159 28154375 10.1038/nrc.2016.145

[embr202152729-bib-0044] Şahin HB, Çelik A (2013) *Drosophila* eye development and photoreceptor specification. eLS 10.1002/9780470015902.a0001147.pub2

[embr202152729-bib-0045] San Juan BP, Andrade‐Zapata I, Baonza A (2012) The bHLH factors Dpn and members of the E(spl) complex mediate the function of Notch signalling regulating cell proliferation during wing disc development. Biol Open 1: 667–676 23213460 10.1242/bio.20121172 PMC3507296

[embr202152729-bib-0046] Siebel C, Lendahl U (2017) Notch signaling in development, tissue homeostasis, and disease. Physiol Rev 97: 1235–1294 28794168 10.1152/physrev.00005.2017

[embr202152729-bib-0047] Sjöqvist M, Andersson ER (2017) Do as I say, Not(ch) as I do: Lateral control of cell fate. Dev Biol 447: 58–70. 28969930 10.1016/j.ydbio.2017.09.032

[embr202152729-bib-0048] Sprinzak D, Lakhanpal A, LeBon L, Santat LA, Fontes ME, Anderson GA, Garcia‐Ojalvo J, Elowitz MB (2010) Cis‐interactions between Notch and Delta generate mutually exclusive signalling states. Nature 465: 86–90 20418862 10.1038/nature08959 PMC2886601

[embr202152729-bib-0049] Suckling RJ, Korona B, Whiteman P, Chillakuri C, Holt L, Handford PA, Lea SM (2017) Structural and functional dissection of the interplay between lipid and Notch binding by human Notch ligands. EMBO J 36: 2204–2215 28572448 10.15252/embj.201796632 PMC5538765

[embr202152729-bib-0050] Thomas U, Speicher SA, Knust E (1991) The *Drosophila* gene Serrate encodes an EGF‐like transmembrane protein with a complex expression pattern in embryos and wing discs. Development 111: 749–761 1840519 10.1242/dev.111.3.749

[embr202152729-bib-0051] Thurmond J, Goodman JL, Strelets VB, Attrill H, Gramates LS, Marygold SJ, Matthews BB, Millburn G, Antonazzo G, Trovisco V*et al* (2019) FlyBase 2.0: the next generation. Nucleic Acids Res 47: D759–D765 30364959 10.1093/nar/gky1003 PMC6323960

[embr202152729-bib-0052] Vässin H, Campos‐Ortega JA (1987) Genetic analysis of delta, a neurogenic gene of *Drosophila* melanogaster. Genetics 116: 433–445 17246393 10.1093/genetics/116.3.433 PMC1203155

[embr202152729-bib-0053] Verdaguer N, Corbalan‐Garcia S, Ochoa WF, Fita I, Gómez‐Fernández JC (1999) Ca(2+) bridges the C2 membrane‐binding domain of protein kinase Calpha directly to phosphatidylserine. EMBO J 18: 6329–6338 10562545 10.1093/emboj/18.22.6329 PMC1171696

[embr202152729-bib-0054] Xu T, Rubin GM (1993) Analysis of genetic mosaics in developing and adult *Drosophila* tissues. Development 117: 1223–1237 8404527 10.1242/dev.117.4.1223

