# Supplementary material for: The conserved C2 phospholipid‐binding domain in Delta contributes to robust Notch signalling
Source: EMBO Rep. 2021 Aug 4;22(10):e52729. doi: 10.15252/embr.202152729 (PMC8490980; doi:10.15252/embr.202152729)
Supplement: Supplementary file 1 — Expanded View Figures PDF [file EMBR-22-e52729-s002.pdf]

Expanded View Figures

Figure EV1. Individual structures and expression and purification of the Dl variants and Notch *in vitro*.

A Comparison of β1-2 loop sequences from *Drosophila* and human Notch ligands. Disulphide bond connectivity within this region is represented by the yellow brackets.

B–E The crystallographic asymmetric units are shown for the three structures (B) Delta—one copy of C2-DSL-EGF1 construct; (C) Serrate—two copies of C2-DSL-EGF1-EGF2 construct; (D) C2 domain of *Drosophila* Delta (rainbow colouring) overlaid on the same region of *Drosophila* Serrate (grey). Disulphide bonds are shown as yellow sticks and the bound calcium ion in the Serrate structure as a grey sphere. The β1-2 loop (blue for Delta, at top) adopts a different conformation in Delta than in Serrate despite conservation of the two stabilizing disulphides; (E) Notch—one copy of EGF11-13. All proteins are shown in a rainbow cartoon representation coloured from blue at the N terminus of the construct to red at the C terminus, except Serrate in D (grey). In B, C, E, Glycosylation is shown in a stick representation as are the side chains to which the glycans are attached and disulphide bonds. Figure drawn using PyMol (The PyMOL Molecular Graphics System, version 2.0 Schrödinger, LLC.)

F Monomeric *Drosophila* Notch ligand (N-EGF3) and receptor (EGF11-13) fragments were expressed in S2 cells as C-terminal His-tagged fusion proteins, purified by IMAC and SEC and visualized by reducing (R) and non-reducing (NR) 10% SDS–PAGE. The Dl variants expressed for *in vitro* experiments were Dl^{WT} as control, $Dl^{\Delta\beta1\text{-}2}$ where the β1-2 loop was deleted, $Dl^{\beta1\text{-}2\_5AA}$ where the β1-2 loop was mutated to 5 alanines. Dl^{F204A} was used as a negative control as this variant reduces Notch binding at Site 2, by altering a key residue within the ligand DSL domain. Additional weak bands in the $Dl^{\Delta\beta1\text{-}2}$ sample, detected in both non-reducing and reducing conditions, indicate carry through of slight impurities.

© 2021 The Authors

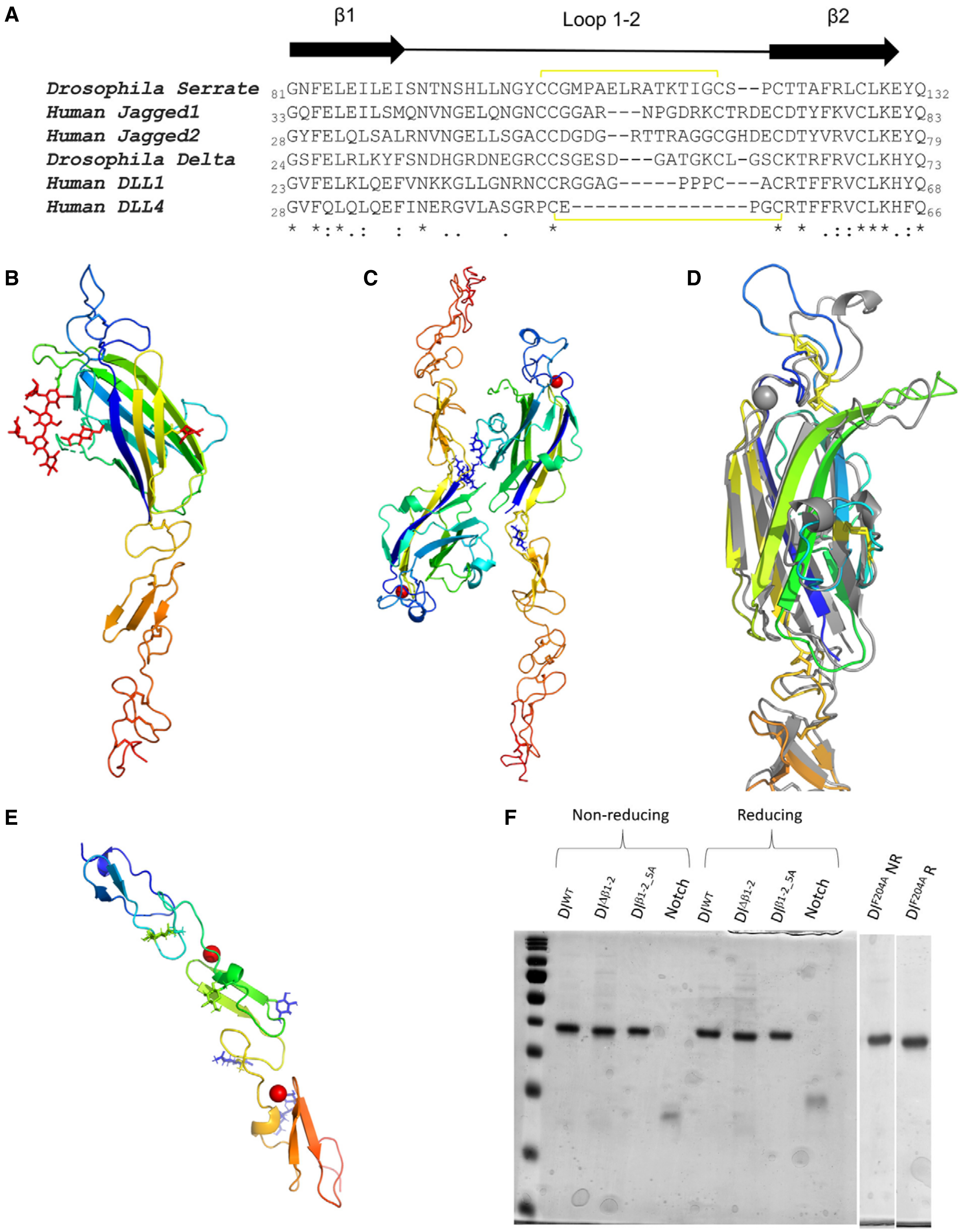

Figure EV1.

 © 2021 The Authors

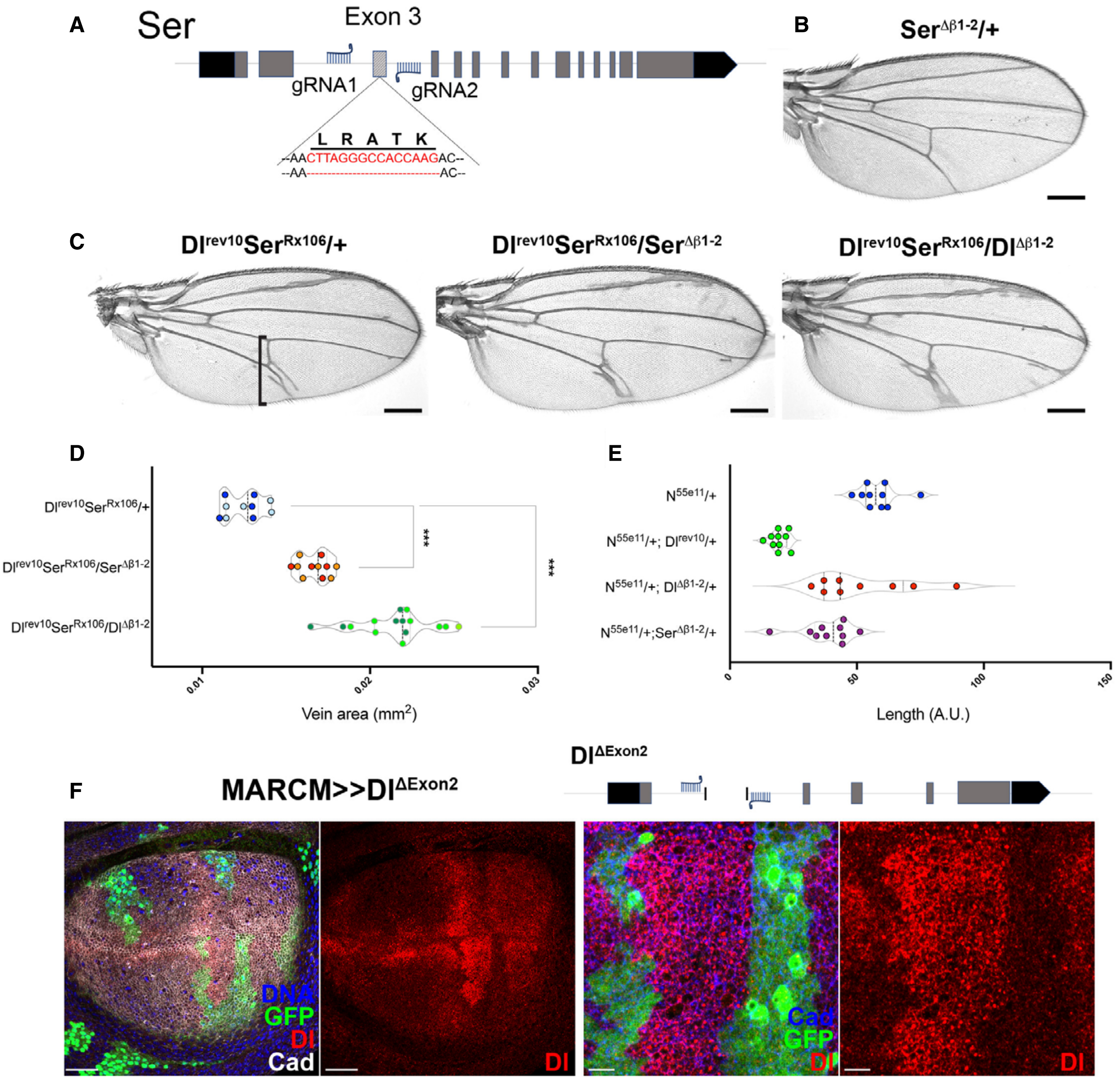

Figure EV2. Ser β1-2 loop mutant enhances vein phenotypes induced by reduced Dl and Ser activities.

A Strategy to remove β1-2 loop sequence from Exon 3 in *Serrate*. Red lettering highlights the sequence of the β1-2 loop of Ser.

B Adult wings from $Ser^{\Delta\beta1\text{-}2}$ heterozygotes have no visible phenotype.

C Wings from $Dl^{\Delta\beta1\text{-}2}$ and $Ser^{\Delta\beta1\text{-}2}$ alleles in combination with Dl^{rev10}; Ser^{Rx106}; both mutants enhance vein thickening. Black square bracket indicates the region used for vein thickness quantification.

D Quantification of vein thickening in wings from females of indicated genotypes; ***$P < 0.0001$ (unpaired *t*-test). Light, dark shading indicates data points from two independent replicates.

E Quantification of L5 vein width at intersection with the wing margin of indicated genotypes; Dl^{rev10} strongly suppressed the small "delta" produced by N^{55e11}/+ ($P < 0.0001$, one-way ANOVA), combinations with $Dl^{\Delta\beta1\text{-}2}$ and $Ser^{\Delta\beta1\text{-}2}$ resulted in mild and variable suppression of borderline significance (ns and $P < 0.05$, respectively, one-way ANOVA). On the violin plots, dashed lines represent the median and the dotted lines show the quartiles.

F Gene diagram showing that the two gRNAs used to replace the Dl Exon 2 also generated a novel allele where the Exon2 was removed, $Dl^{\Delta Exon2}$. $Dl^{\Delta Exon2}$ mutant clones (green) in wing imaginal discs stained for Dl (red) and the apical marker Cadherin (blue); Dl protein is absent in the mutant cells, indicating that little or no stable protein is made most likely because the signal sequence has been deleted.

Data information: Scale bars represent 200 μm (B, C), 50 μm (F) and 10 μm in the magnified image of F.

© 2021 The Authors

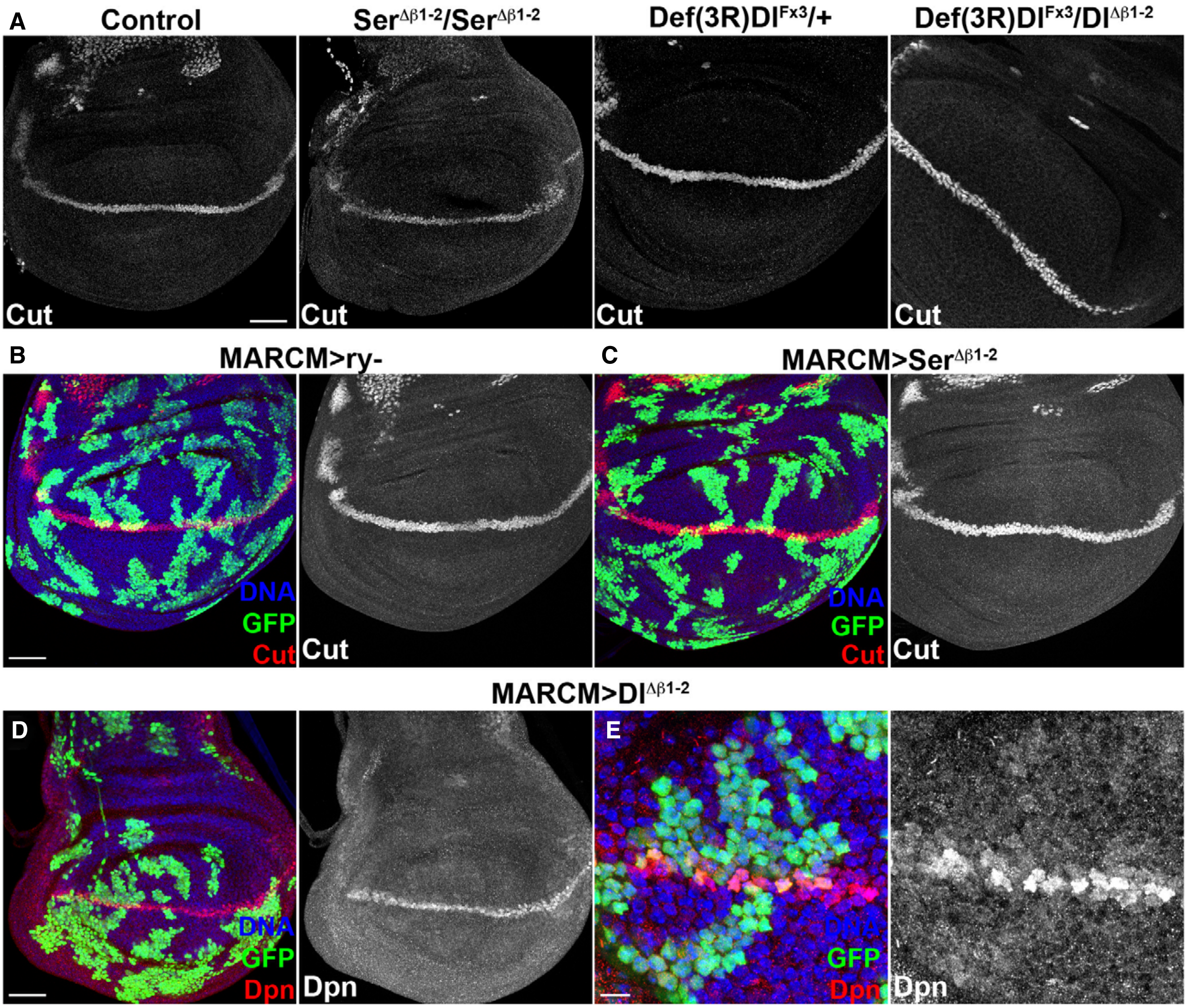

Figure EV3. Expression of Notch targets *cut* and *dpn* is unaffected by $Dl^{\Delta\beta1\text{-}2}$ or $Ser^{\Delta\beta1\text{-}2}$.

A Expression of Cut at the wing dorsal-ventral (DV) boundary is unperturbed in wing discs of the genotypes indicated.

B, C Cut (red) expression is similar in wild-type (MARCM > *ry*, green, (B) or $Ser^{\Delta\beta1\text{-}2}$ homozygous (MARCM > $Ser^{\Delta\beta1\text{-}2}$, green, (C) clones that intersect the DV boundary. Individual Cut expression for each experiment is shown in grayscale on the right subpanels.

D, E $Dl^{\Delta\beta1\text{-}2}$ homozygous clones (green) that intersect the DV boundary retain expression of Dpn (red), higher magnification in (E). Individual Dpn expression is shown in grayscale on the right subpanels.

Data information: Scale bars: 50 μm (A–D) or 10 μm (E).

 © 2021 The Authors

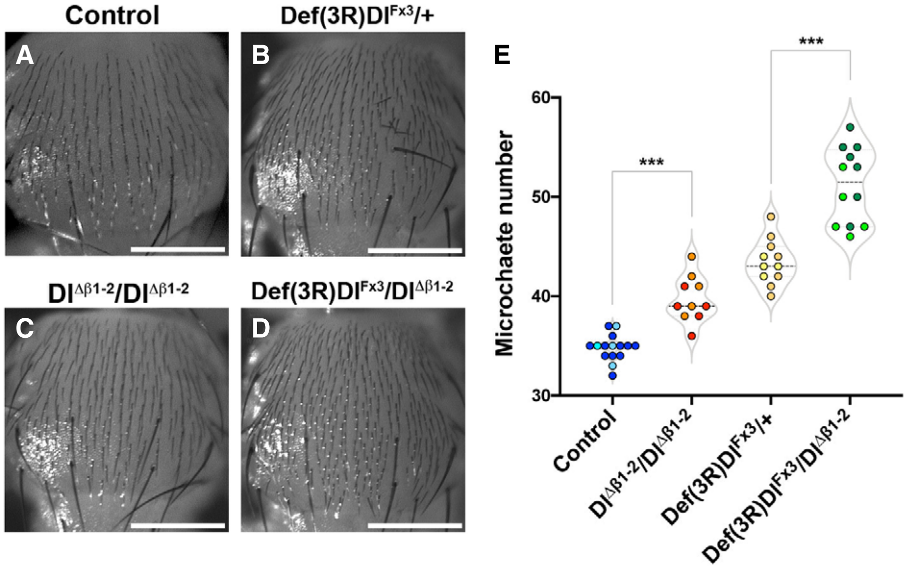

Figure EV4. ***$Dl^{\Delta\beta1\text{-}2}$* compromises signalling during microchaete selection.**

A–D Microchaete distribution in the notum of adult flies of the following genotypes: (A) Control, *yw*; (B) *Df(3R)Dl^{Fx3}/+*; (C) *$Dl^{\Delta\beta1\text{-}2}/Dl^{\Delta\beta1\text{-}2}$*; (D) *Df (3R)Dl^{Fx3}/Dl$^{\Delta\beta1\text{-}2}$*. Homozygous *$Dl^{\Delta\beta1\text{-}2}$* mutants and *$Dl^{\Delta\beta1\text{-}2}$* combined with *Df(3R)Dl^{Fx3}* increases the number of the microchaetes in the notum.

E Quantification of microchaete numbers which were scored in a central region (white rectangle in Fig 2D), ***$P < 0.0001$ (unpaired *t*-test). Each dot represents an individual fly, and light or dark shading indicates data points from two independent crosses. On the violin plot, dashed line represents the median and the dotted lines show the quartiles.

Data information: Scale bars (A–D) correspond to 500 μm.

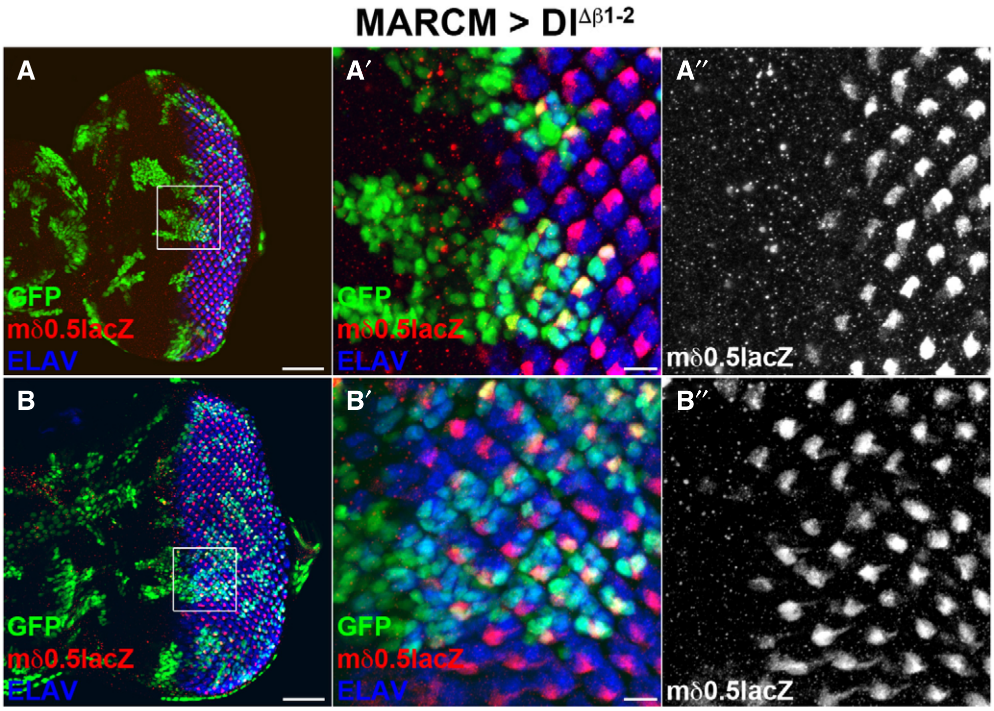

Figure EV5. ***E(spl)mδ0.5* expression is retained in homozygous *$Dl^{\Delta\beta1\text{-}2}$* clones.**

A *$Dl^{\Delta\beta1\text{-}2}$* homozygous clones (green) in eye imaginal discs with *E(spl)mδ0.5* (red) reporter expression, ELAV (blue) marks all photoreceptors. (A'A'') Higher magnification of the equatorial region (marked by white square in A), *E(spl)mδ0.5* (red, A'; white, A'') is detected in R4 of wild-type and mutant clusters, including clusters where R3 is mutant.

B Large homozygous *$Dl^{\Delta\beta1\text{-}2}$* clone (green), *E(spl)mδ0.5* (red) expression is unaffected, ELAV (blue) marks all photoreceptors. (B', B'') Higher magnification of the equatorial region (marked by white square in B), *E(spl)mδ0.5* (red, B'; white, B'') is detected in R4 of wild-type and mutant clusters, including clusters where R3 is mutant.

Data information: Scale bars correspond to 50 μm (A, B) or 10 μm (A', B').

© 2021 The Authors
