# Supplementary material for: The conserved C2 phospholipid‐binding domain in Delta contributes to robust Notch signalling
Source: EMBO Rep. 2021 Aug 4;22(10):e52729. doi: 10.15252/embr.202152729 (PMC8490980; doi:10.15252/embr.202152729)
Supplement: Supplementary file 2 — Table EV1 [file EMBR-22-e52729-s003.docx]

**Table EV1 - Primers list**

| **Primer name** | **Sequence 5’ to 3’** |
| --- | --- |
|  | |
| ***Generation of the gRNAs plasmids*** | |
| PCFD4_Dl Ex2 gRNA1 | TATATAGGAAAGATATCCGGGTGAACTTCGCTTCAAAACGAAAGTCGAGGTTTTAGAGCTAGAAATAGCAAG |
| PCFD4_Dl Ex2 gRNA2 | ATTTTAACTTGCTATTTCTAGCTCTAAAACTGTTGTCTTCTTTATACGTCGACGTTAAATTGAAAATAGGTC |
| PCFD4_Ser Ex3 gRNA1 | TATATAGGAAAGATATCCGGGTGAACTTCGGAATGTAAGCTGGGCAGGGGTTTTAGAGCTAGAAATAGCAAG |
| PCFD4_Ser Ex3 gRNA2 | ATTTTAACTTGCTATTTCTAGCTCTAAAACCGTAAAAATGCAGTCTTACCGACGTTAAATTGAAAATAGGTC |
|  |  |
| ***Generation of the HR donor plasmids*** | |
| Delta HA1 Fw | CGGGGCCGAATTCGCAGGTGGCAGTATTAATTAATCTGTTTAATAAGAAGCTAA |
| Delta HA1 Rev | GTTAAGATCGCAGGTGCTCGACTTTCGTTTTGAAGCGC |
| Delta Exon2 Fw | CTATCTTTCTAGGGTTAAGGAAAGCCAAGCCAAGTGGAAT |
| Delta Exon2 Rev | AACGTATAAAGAAGACAACAAAATATTTATTGACTGACTGAATGAG |
| Delta HA2 Fw | TCAGTCAGTCAATAAATATTTTGTTGTCTTCTTTATACGTTGAG |
| Delta HA2 Rev | TCTTTTCCCGGGGAAGAGCGTTATTGCTACTGGCATAAATACTT |
| pHD_DsRed_Dl bb 1 Fw | ATTTATGCCAGTAGCAATAACGCTCTTCCCCGGGAAAAGA |
| pHD_DsRed_Dl bb 1 Rev | ATTCCACTTGGCTTGGCTTTCCTTAACCCTAGAAAGATAGTCTGC |
| pHD_DsRed_Dl bb 2 Fw | GCGCTTCAAAACGAAAGTCGAGCACCTGCGATCTTAACC |
| pHD_DsRed_Dl bb 2 Rev | ACAGATTAATTAATACTGCCACCTGCGAATTCGGCCCCGA |
| Serrate HA1 Fw | CGAATTCGCAGGTGGCTTTGAGGCCCAAACAAAAGGA |
| Serrate HA1 Rev | TAAGATCGCAGGTGCTCCCTGCCCAGCTTACATTC |
| Serrate Exon3 Fw | TTCTAGGGTTAAGGAAATTGGCTCAGAGGGGAGGG |
| Serrate Exon3 Rev | AGACTGCATTTTTACGTCTTAACTTTCTTTGACAAGCAAAAGGCG |
| Serrate HA2 Fw | TGCTTGTCAAAGAAAGTTAAGACGTAAAAATGCAGTCTTACTTCGAC |
| Serrate HA2 Rev | TTCCCGGGGAAGAGCGGCCCCATGATTCTGGTGC |
| pHD_DsRed_Ser bb 1 Fw | ACCAGAATCATGGGGCCGCTCTTCCCCGGGAA |
| pHD_DsRed_Ser bb 1 Rev | TCCCCTCTGAGCCAATTTCCTTAACCCTAGAAAGATAGTCTGC |
| pHD_DsRed_Ser bb 2 Fw | TGTAAGCTGGGCAGGGAGCACCTGCGATCTTAACC |
| pHD_DsRed_Ser bb 2 Rev | TTGTTTGGGCCTCAAAGCCACCTGCGAATTCGG |
|  |  |
| ***Mutation of the β1-2 loops*** | |
| Deletion of Dl β1-2 loop Fw | TGCCTGGGCAGCTGC |
| Deletion of Dl β1-2 loop Rev | GTCCGACTCCCCGCT |
| Deletion of Ser β1-2 loop Fw | ACGATAGGTAAGTCCATCTTTAAGTTTATTAAAGCCC |
| Deletion of Ser β1-2 loop Rev | TTCCGCTGGCATGCCG |
|  |  |
| ***Sequencing of the transformants*** | |
| Dl Ex2 seq | GTTCACAGTTCCGGC |
| Dl UP5 Fw | TGCCTGTTGCTGCTGCTTC |
| Dl 3Down Rev | AGGGAGAGGGATCGAGATAGAGG |
| Ser Up5 Fw | TAGTAAGCAGCAAGAACCGGT |
| Ser 3Down Rev | ATCTCCGCAGACCGAGATGA |
| Transp Rev | CAGACCGATAAAACACATGCGTC |
| Transp Fw | ATGCACAGCGACGGATTCG |
|  |  |
| ***Protein expression*** | |
| Delta β1-2 deletion Fw | AGCGGAGAGAGTGATTGCCTGGGCAGTTGCAAGAC |
| Delta β1-2 deletion Rev | GCAACTGCCCAGGCAATCACTCTCTCCGCTGCAGC |
| Serrate β1-2 deletion Fw | GCGGCATGCCAGCGGAAACGATAGGCTGCTCGCCATGC |
| Serrate β1-2 deletion Rev | GGCGAGCAGCCTATCGTTTCCGCTGGCATGCCGCAGC |
| Delta F204A Fw | GACGATAGCGCCGGCCACAGTACCTGCAG |
| Delta F204A Rev | GTGGCCGGCGCTATCGTCGCGTGGGCG |
| pExS2-2fusion Fw | GAAGCTTGGTACCCTCGAGCTCAGCT |
| pExS2-2fusion Rev | CAAACTAAGATTTAGTCAGATATCGATCGATCC |
