# Supplementary material for: The conserved C2 phospholipid‐binding domain in Delta contributes to robust Notch signalling
Source: EMBO Rep. 2021 Aug 4;22(10):e52729. doi: 10.15252/embr.202152729 (PMC8490980; doi:10.15252/embr.202152729)
Supplement: Supplementary file 3 [file EMBR-22-e52729-s001.pdf]

EMBO reports

The conserved C2 phospholipid-binding Domain in Delta contributes to robust Notch signalling

Torcato Martins, Yao Meng, Boguslawa Korona, Richard Suckling, Stephen Johnson, Penny Handford, Susan Lea, and Sarah J. Bray
DOI: 10.15252/embr.202152729

Corresponding author(s): Sarah J. Bray (sjb32@cam.ac.uk) , Susan Lea (susan.lea@path.ox.ac.uk), Penny Handford (penny.handford@bioch.ox.ac.uk)

Review Timeline:

Submission Date:	22nd Feb 21
Editorial Decision:	14th May 21
Editor Correspondence:	20th May 21
Revision Received:	7th Jun 21
Editorial Decision:	2nd Jul 21
Revision Received:	10th Jul 21
Accepted:	15th Jul 21

Editor: Martina Rembold

Transaction Report:

(Note: With the exception of the correction of typographical or spelling errors that could be a source of ambiguity, letters and reports are not edited. Depending on transfer agreements, referee reports obtained elsewhere may or may not be included in this compilation. Referee reports are anonymous unless the Referee chooses to sign their reports.)

1st Editorial Decision 14th May 2021

Dear Prof. Bray

Thank you for the submission of your manuscript to EMBO reports. Please accept my apologies for the unusual delay in handling your manuscript. Unfortunately, one referee was not responsive and I had to find a replacement for the evaluation of the structural aspect of your study, which is now underway.

So far, we have received two referee reports that are copied below. Given that both referees are in fair agreement that you should be given a chance to revise the manuscript, I would like to ask you to begin revising your study along the lines suggested by the referees. Please note that this is a preliminary decision made in the interest of time, and that it is subject to change should the third referee offer very strong and convincing reasons for this. Once we received the final report on your manuscript, I will forward it to you as well.

Please address all referee concerns in the manuscript and in a complete point-by-point response and take their suggestions on board. Acceptance of the manuscript will depend on a positive outcome of a second round of review. It is EMBO reports policy to allow a single round of revision only and acceptance or rejection of the manuscript will therefore depend on the completeness of your responses included in the next, final version of the manuscript.

We invite you to submit your manuscript within three months of a request for revision. This would be August 14th in your case. However, we are aware of the fact that many laboratories are not fully functional due to COVID-19 related shutdowns and we have therefore extended the revision time for all research manuscripts under our scooping protection to allow for the extra time required to address essential experimental issues. Please contact us to discuss the time needed and the revisions further.

***IMPORTANT NOTE: we perform an initial quality control of all revised manuscripts before re-review. Your manuscript will FAIL this control and the handling will be DELAYED if the following APPLIES:

1) A data availability section is missing.
2) Your manuscript contains error bars based on n=2. Please use scatter blots showing the individual datapoints in these cases. The use of statistical tests needs to be justified.

When submitting your revised manuscript, please carefully review the instructions that follow below. Failure to include requested items will delay the evaluation of your revision.***

When submitting your revised manuscript, we will require:

1) a .docx formatted version of the manuscript text (including legends for main figures, EV figures and tables). Please make sure that the changes are highlighted to be clearly visible.

2) individual production quality figure files as .eps, .tif, .jpg (one file per figure).
Please download our Figure Preparation Guidelines (figure preparation pdf) from our Author Guidelines pages

https://www.embopress.org/page/journal/14693178/authorguide for more info on how to prepare your figures.

3) a .docx formatted letter INCLUDING the reviewers' reports and your detailed point-by-point responses to their comments. As part of the EMBO Press transparent editorial process, the point-by-point response is part of the Review Process File (RPF), which will be published alongside your paper.

4) a complete author checklist, which you can download from our author guidelines (). Please insert information in the checklist that is also reflected in the manuscript. The completed author checklist will also be part of the RPF.

5) Please note that all corresponding authors are required to supply an ORCID ID for their name upon submission of a revised manuscript (). Please find instructions on how to link your ORCID ID to your account in our manuscript tracking system in our Author guidelines
()

6) We replaced Supplementary Information with Expanded View (EV) Figures and Tables that are collapsible/expandable online. A maximum of 5 EV Figures can be typeset. EV Figures should be cited as 'Figure EV1, Figure EV2" etc... in the text and their respective legends should be included in the main text after the legends of regular figures.

- For the figures that you do NOT wish to display as Expanded View figures, they should be bundled together with their legends in a single PDF file called *Appendix*, which should start with a short Table of Content. Appendix figures should be referred to in the main text as: "Appendix Figure S1, Appendix Figure S2" etc. See detailed instructions regarding expanded view here:

- Additional Tables/Datasets should be labeled and referred to as Table EV1, Dataset EV1, etc. Legends have to be provided in a separate tab in case of .xls files. Alternatively, the legend can be supplied as a separate text file (README) and zipped together with the Table/Dataset file.

7) Please list the accession codes for the structural data in a separate Data availability section that follows the model below and please remember to provide a reviewer password if the datasets are not yet public (see also <
https://www.embopress.org/page/journal/14693178/authorguide#dataavailability>).
Data availability

The datasets (and computer code) produced in this study are available in the following databases:

- RNA-Seq data: Gene Expression Omnibus GSE46843
(https://www.ncbi.nlm.nih.gov/geo/query/acc.cgi?acc=GSE46843)
- [data type]: [name of the resource] [accession number/identifier/doi] ([URL or identifiers.org/DATABASE:ACCESSION])

*** Note - All links should resolve to a page where the data can be accessed. ***

8) We would also encourage you to include the source data for figure panels that show essential data. Numerical data should be provided as individual .xls or .csv files (including a tab describing the data). For blots or microscopy, uncropped images should be submitted (using a zip archive if

multiple images need to be supplied for one panel). Additional information on source data and instruction on how to label the files are available .

9) Our journal encourages inclusion of *data citations in the reference list* to directly cite datasets that were re-used and obtained from public databases. Data citations in the article text are distinct from normal bibliographical citations and should directly link to the database records from which the data can be accessed. In the main text, data citations are formatted as follows: "Data ref: Smith et al, 2001" or "Data ref: NCBI Sequence Read Archive PRJNA342805, 2017". In the Reference list, data citations must be labeled with "[DATASET]". A data reference must provide the database name, accession number/identifiers and a resolvable link to the landing page from which the data can be accessed at the end of the reference. Further instructions are available at .

10) Regarding data quantification
The following points must be specified in each figure legend:
- the name of the statistical test used to generate error bars and P values,
- the number (n) of independent experiments (please specify technical or biological replicates) underlying each data point,
- the nature of the bars and error bars (s.d., s.e.m.)
Discussion of statistical methodology can be reported in the materials and methods section, but figure legends should contain a basic description of n, P and the test applied.
- Please also include scale bars in all microscopy images.

11) As part of the EMBO publication's Transparent Editorial Process, EMBO reports publishes online a Review Process File to accompany accepted manuscripts. This File will be published in conjunction with your paper and will include the referee reports, your point-by-point response and all pertinent correspondence relating to the manuscript.

You are able to opt out of this by letting the editorial office know (emboreports@embo.org). If you do opt out, the Review Process File link will point to the following statement: "No Review Process File is available with this article, as the authors have chosen not to make the review process public in this case."

We would also welcome the submission of cover suggestions, or motifs to be used by our Graphics Illustrator in designing a cover.

I look forward to seeing a revised version of your manuscript when it is ready. Please let me know if you have questions or comments regarding the revision.

Kind regards,

Martina Rembold, PhD
Senior Editor
EMBO reports

****

Referee #1:

Manuscript entitled "Delta C2 Domain ß1-2 loop contributes to robust Notch signaling" by Martins

et al. (manuscript EMBOR-2021-52729-T)

In this manuscript Martin et al. investigate the meaning of the However the importance for the activity of the ligands remained unclear. The authors here use the Drosophila model to resolve this issue. They show that the C2 domain is also present in the Drosophila ligands and that it is a functional C2 domain, as it can bind to liposomes. The demonstration of the existence of a C2 domain in the Drosophila DSL ligands, opened up the chance to perform thorough in vivo studies of its function. To do so the authors generated alleles by genome editing that lack the ß1-2 loop of the C2 domain, which, as they show, is essential for lipid binding. These alleles exhibited weak phenotypes in homozygosity. Especially, the further study of the Dl[illegible]ß1-2 allele revealed that the ß1-2 loop is important for the full activity of the DSL ligands.
The presented results are based on solid state of the art experiments and produced results that are important for the whole community of Notch signaling, as they answer an important question lingering in the field for some time. Hence, I strongly support the publication in EMBO reports after taking in account the minor point listed below.

Minor point: the authors state that the trafficking of the Dl[illegible]ß1-2 is unaltered. I think they should tune down this statement, since the presented experiments are of qualitative nature and a quantification of endocytosis or endosomal trafficking has not been shown. I know that this is difficult therefore I suggest to write that the endosomal trafficking is not grossly affected.

Referee #3:

1. Does this manuscript report a single key finding?

Yes. The manuscript extends previous work on the lipid-binding C2 region of Notch ligands, examining the structure of these region in the Drosophila ligands Delta and Serrate, demonstrating the effects of these regions on lipid binding in vitro, and demonstrating in vivo Notch signaling defects caused by removal of one of these regions.

2. Is the reported work of significance (YES), or does it describe a confirmatory finding or one that has already been documented using other methods or in other organisms etc ?

Yes, this is a novel analysis of the Drosophila Notch ligands, and with careful in vivo tests of the function of the lipid-binding regions of Notch ligands, confirming and extending previously published in vitro signaling assays.

3. Is it of general interest to the molecular biology community?

Yes, this is a novel finding on a critical signaling pathway.

4. Is the single major finding robustly documented using independent lines of experimental evidence (YES), or is it really just a preliminary report requiring significant further data to become convincing, and thus more suited to a longer¬format article (NO)?

Yes.

Review:

The manuscript extends previous work on the lipid-binding C2 region of Notch ligands. Previous work has examined the structure of this N-terminal portion of the ligands and demonstrated that it binds lipids. Mutation of these regions in vertebrate Notch ligands reduces lipid binding and Notch signaling in vitro without detectably altering binding to Notch. The manuscript extends the published studies by examining the structure of these region in the Drosophila ligands Delta and Serrate. The manuscript shows that deleting one of the lipid-binding loops from the C2 region reduces lipid binding in vitro, similar to published work using vertebrate ligands. Finally, the manuscript shows that CRISPR-mediated removal of this loop from the endogenous Drosophila Delta and Serrate genes results in vivo defects. The defects in homozygotes are consistent with a mild loss of Notch signaling, and the Delta loop mutant strengthens the dominant defects caused by Delta null alleles. Intriguingly, as with null Delta alleles, heterozygosity for the Delta loop allele weakens the dominant wing notching phenotype caused by a Notch null allele. The authors suggest that this gain in Notch signaling is caused by modulating the level or effectiveness of inhibitory "cis" interactions between the Notch and Delta expressed on the same cell.

The findings for the most part are solid and well-supported, and will be of high interest to the many researchers working on Notch signaling, as well as those interested in the role of lipid-binding domains.

1. It would help the reader if the authors briefly explained why the picked the beta1-2 loop for their mutational analyses, of the several loops described in the structural analysis.

2. Previous studies found that reducing a ligand's lipid binding did not affect its binding to Notch. Here the authors say that "the variant with the loop deletion...retained Notch binding", without analyzing or commenting on any change in that binding. However, in Fig 1E the binding of the Delta loop mutant to Notch is reduced. While the authors provide no statistical analysis of this difference, it looks likely to be significant. This would suggest that the Notch-binding and lipid-binding regions of Delta are not quite as independent as the authors say, which would modify their proposed mechanisms.

3. The authors only describe the genetic interaction between Notch and Delta loop mutants in one context, the notching of the wing margin. Are there other phenotypes that suggest a similar increase in Notch signaling, or is it limited to the margin?

4. While the gain of Notch signaling is consistent with reduced inhibitory cis interactions, a more stringent test for cis interactions in vivo is to place ligand mutant cells next to normal cells and look for increased signaling only in the mutant cells. While this might not work if the effect is too weak, I am nonetheless curious about whether the authors have looked at Delta loop mutant clones in either wild type or Notch heterozygote wing discs?

5. The Discussion does not mention the finding that the Delta loop mutant increases Notch signaling in a Notch heterozygote. Instead, the authors only discuss the reduced Notch signaling, and in fact suggest that "Loss of interactions with certain types of lipids might bias how the ligand interacts with the receptor in favour of inhibition", suggesting in this context increased cis inhibition. Yet in the Results they imply that the loop mutant "modulates" cis inhibition in a way that increases signaling, at least in the context of the wing margin. I would prefer a more careful discussion of this issue.

6. Minor points, typos:

pg. 4 "PC:PS:PE-" The abbreviation should be explained, either here, the legend or the methods.

pg. 5 deltaExon2- Does this cause a frameshift or does it make the rest of the protein? This detail could be added to the EV2 legend or methods.

pg. 6- "expression of genes cut and deadpan" - References?

pg. 7- "when they are present on the same surface"- Reference Micchelli et al '97?

pg. 7- "supressed" - suppressed

pg. 7 "when combined with a Delta loss of function allele" Mention the data in the figure, especially if this is a new result. If this is an old result, what's the reference?

pg. 10- "results coupling C2 domain lipid binding to Notch binding"- What kind of coupling are the authors referring to?

Dear Sarah,

We have now received the final report for your manuscript EMBOR-2021-52729-T, 'Delta C2 Domain beta1-2 loop contributes to robust Notch signalling'. I have copied it below my signature.

Also this referee is very positive and supports publication after rather minor revisions. Please also address the concerns from referee #4 in your manuscript and point-by-point response.

Kind regards,
Martina

Martina Rembold, PhD
Senior Editor
EMBO reports

****

Referee #4

This paper uses structural studies as well as experiments in Drosophila to understand the role of the N-terminal C2 domain of Notch ligands in signalling. It expands on previous work by the authors (Suckling et al. 2017) that indicates the C2 domain is involved in lipid binding.
The document is well written and easy to follow. The paper describes three new crystal structures. These are N-terminal fragments of Drosophila Notch ligands Delta and Serrate, alongside EGFs11-13 of Drosophila Notch, a region known to interact with ligand. As expected, these structures are very similar to their human counterparts. Their in vivo experiments using Drosophila show that deletion of 5 amino acids from a loop in the C2 domain of Delta (located away from the known Notch interaction site) results in phenotypes consistent with disrupted Notch signalling. Although the work does not directly link the C2 domain to phospholipid binding in vivo, it adds further evidence to the hypothesis that that the C2 domain fine-tunes Notch signalling through interactions with the cell membrane.

Commenting specifically, as requested, on evaluation of the structural aspects of the data (Fig. 1, EV1, Table1).
The data collection and refinement statistics presented in Table 1 appear sound for all three structures presented (PDB validation reports and examples of electron density were not provided). I have a few minor comments that would improve the analysis.
-The β1-2 loop in human Delta-like and Jagged ligands is flanked by two cysteines that form a disulphide. These are also present in Delta and Serrate. These have been shown in the figures but not mentioned. The authors could add a line to the figure legend remarking on these.
-Delta is compared to Dll4 and Serrate is compared to Jagged-1. However Dll4 does not have a significant β 1-2 loop, and thus it might be useful to show how Delta compares to Jagged-1 (and Jagged 2) which both have longer loops.
-A sequence alignment highlighting the loops in the C2 domain for Delta/Serrate and human DLL1/DLL4/Jag1/Jag2 proteins would also be a valuable addition.

EMBOR-2021-52729-T: Response to reviewers.

Changes to the text that have been introduced to address the comments from the reviewer are in red font in the manuscript file.

Referee #1:

In this manuscript Martin et al. investigate the meaning of the However the importance for the activity of the ligands remained unclear. The authors here use the Drosophila model to resolve this issue. They show that the C2 domain is also present in the Drosophila ligands and that it is a functional C2 domain, as it can bind to liposomes. The demonstration of the existence of a C2 domain in the Drosophila DSL ligands, opened up the chance to perform thorough in vivo studies of its function. To do so the authors generated alleles by genome editing that lack the ß1-2 loop of the C2 domain, which, as they show, is essential for lipid binding. These alleles exhibited weak phenotypes in homozygosity. Especially, the further study of the Dl∆ß1-2 allele revealed that the ß1-2 loop is important for the full activity of the DSL ligands.
The presented results are based on solid state of the art experiments and produced results that are important for the whole community of Notch signaling, as they answer an important question lingering in the field for some time. Hence, I strongly support the publication in EMBO reports after taking in account the minor point listed below.
We are glad that the reviewer considers our results important for the field.

Minor point: the authors state that the trafficking of the Dl∆ß1-2 is unaltered. I think they should tune down this statement, since the presented experiments are of qualitative nature and a quantification of endocytosis or endosomal trafficking has not been shown. I know that this is difficult therefore I suggest to write that the endosomal trafficking is not grossly affected.
We agree with the reviewer that we cannot rule out a subtle change to endocytosis. We have modified the text accordingly (**page 9** final sentence of results "*its trafficking following endocytic uptake is not grossly affected although we cannot rule out a subtle change*")

Referee #3:

1. Does this manuscript report a single key finding? Yes. The manuscript extends previous work on the lipid-binding C2 region of Notch ligands, examining the structure of these region in the Drosophila ligands Delta and Serrate, demonstrating the effects of these regions on lipid binding in vitro, and demonstrating in vivo Notch signaling defects caused by removal of one of these regions.
2. Is the reported work of significance (YES), or does it describe a confirmatory finding or one that has already been documented using other methods or in other organisms etc ? Yes, this is a novel analysis of the Drosophila Notch ligands, and with careful in vivo tests of the function of the lipid-binding regions of Notch ligands, confirming and extending previously published in vitro signaling assays.
3. Is it of general interest to the molecular biology community? Yes, this is a novel finding on a critical signaling pathway.
4. Is the single major finding robustly documented using independent lines of experimental evidence (YES), or is it really just a preliminary report requiring significant further data to become convincing, and thus more suited to a longer¬format article (NO)? Yes.

Review:
The manuscript extends previous work on the lipid-binding C2 region of Notch ligands. Previous work has examined the structure of this N-terminal portion of the ligands and demonstrated that it binds lipids. Mutation of these regions in vertebrate Notch ligands reduces lipid binding and Notch signaling in vitro without detectably altering binding to Notch. The manuscript extends the published studies by examining the structure of these region in the Drosophila ligands Delta and Serrate. The manuscript shows that deleting one of the lipid-binding loops from the C2 region reduces lipid binding in vitro, similar to published work using vertebrate ligands. Finally, the manuscript shows that CRISPR-mediated removal of this loop from the endogenous Drosophila Delta and Serrate genes results in vivo defects. The defects in homozygotes are consistent with a mild loss of Notch signaling, and the Delta loop mutant strengthens the dominant defects caused by Delta null alleles. Intriguingly, as with null Delta alleles, heterozygosity for the Delta loop allele weakens the dominant wing notching

phenotype caused by a Notch null allele. The authors suggest that this gain in Notch signaling is caused by modulating the level or effectiveness of inhibitory "cis" interactions between the Notch and Delta expressed on the same cell.
The findings for the most part are solid and well-supported, and will be of high interest to the many researchers working on Notch signaling, as well as those interested in the role of lipid-binding domains.
We are glad the reviewer considers our results of high interest to the field. We are grateful for their constructive comments.

1. It would help the reader if the authors briefly explained why the picked the beta1-2 loop for their mutational analyses, of the several loops described in the structural analysis.
The β1-2 loop is one of two loops at the apex of the C2 domain that make a key contribution to C2-mediated lipid binding in other proteins. These two loops, β1-2 and β5-6, were therefore the prime targets to disrupt such binding. Of the two, the β1-2 loop is encoded by a small exon, making it plausible to plan a strategy for the mutagenesis by CRISPR/Cas9 mediated genome engineering. This point has been explained more fully in the results **on page 4 paragraph 2, page 5 final paragraph**.

2. Previous studies found that reducing a ligand's lipid binding did not affect its binding to Notch. Here the authors say that "the variant with the loop deletion...retained Notch binding", without analyzing or commenting on any change in that binding. However, in Fig 1E the binding of the Delta loop mutant to Notch is reduced. While the authors provide no statistical analysis of this difference, it looks likely to be significant. This would suggest that the Notch-binding and lipid-binding regions of Delta are not quite as independent as the authors say, which would modify their proposed mechanisms.
We thank the reviewer for pointing out that we had omitted to explain the slight decrease in the *in vitro* Notch binding from the $Dl^{\Delta\beta1\text{-}2}$ mutation. The Delta constructs are made as secreted fragments that are purified from S2 cells. In the case of $Dl^{\Delta\beta1\text{-}2}$ mutant, a small amount of impurity has been carried through (shown in EV1 (D)). As stated in the legend to this figure, we attribute the small difference in Notch binding to this preparation being slightly less pure than for the wild-type. This binding difference is much less than that observed for a variant which has been engineered (F204A) to be defective in binding to Notch at Site 1. We have now added a statement and our explanation for the slight decrease in the main results (**page 5 paragraph 2**) and a comment in the methods (**page 13 paragraph 2**).

3. The authors only describe the genetic interaction between Notch and Delta loop mutants in one context, the notching of the wing margin. Are there other phenotypes that suggest a similar increase in Notch signaling, or is it limited to the margin?
We appreciate the comments from the referee and agree that we have not shown the effect is a global one. We have focussed our analysis on the wing margin phenotype because the "nick" in the wing margin is the most robust and reproducible phenotype from *Notch/+* (reduced Notch function). It is also one that has been characterized for cis-inhibition, based on both loss and gain of function experiments. However, as mild venation defects in *Notch/+* are also suppressed by *Dl* alleles, we have now assessed the effects of $Dl^{\Delta\beta1\text{-}2}$ on this and found a subtle but variable modification in the same direction, albeit less robust than for a *Dl* null allele. These data have been added to **new Figure EV2** with a comment in the text (**page 7 paragraph 2**). Other contexts where cis-inhibition has been reported include the R6/R7 fate decision in the eye, but this has only been seen with strong loss of function clones and we did not detect a similar signalling reversal with the $Dl^{\Delta\beta1\text{-}2}$ mutations. We have modified the text in the results to explain the limitations **on page 7, end of paragraph 2**, and commented in the discussion that the effects on cis-inhibition may depend on the context and the levels of the different proteins present **(page 10 paragraph 2).**

4. While the gain of Notch signaling is consistent with reduced inhibitory cis interactions, a more stringent test for cis interactions in vivo is to place ligand mutant cells next to normal cells and look for increased signaling only in the mutant cells. While this might not work if the effect is too weak, I am nonetheless curious about whether the authors have looked at Delta loop mutant clones in either wild type or Notch heterozygote wing discs?
We appreciate the suggestions, which are related to those above and illustrate the challenges of clearly demonstrating changes in cis-inhibition. We have analyzed the effects of $Dl^{\Delta\beta1\text{-}2}$ mutant clones in wild type wing and eye discs, as shown in Figures EV3 and EV5. In neither case do we detect any reproducible effect on signaling in the mutant cells, as occurs with strong loss-of-function *Delta* alleles

when, in a few cases, there is upregulation of target genes in the mutant cells. As the reviewer suggests, this requires a very penetrant loss of cis-inhibition and is only detected in few locations (e.g. very close to the d/v boundary in the wing disc). The effects of $Dl^{\Delta\beta1\text{-}2}$ mutant are very mild and not sufficient to perturb the direction of signaling sufficiently. We have added further comments to the results **on page 7, end of paragraph 2**, to make clear the limitations

5. The Discussion does not mention the finding that the Delta loop mutant increases Notch signaling in a Notch heterozygote. Instead, the authors only discuss the reduced Notch signaling, and in fact suggest that "Loss of interactions with certain types of lipids might bias how the ligand interacts with the receptor in favour of inhibition", suggesting in this context increased cis inhibition. Yet in the Results they imply that the loop mutant "modulates" cis inhibition in a way that increases signaling, at least in the context of the wing margin. I would prefer a more careful discussion of this issue.
We thank the reviewer for pointing this out and have modified the discussion accordingly, to take account of the effects on cis-inhibition as well as on activation (**page 10 end of paragraph 2**).

6. Minor points, typos:

pg. 4 "PC:PS:PE-" The abbreviation should be explained, either here, the legend or the methods.
The abbreviations have been clarified in the results and the methods. “Phosphatidylcholine (PC): Phosphatidylserine (PS): Phosphatidylethanolamine-fluoroscein (PE) in an 80:15:5 ratio”

pg. 5 deltaExon2- Does this cause a frameshift or does it make the rest of the protein? This detail could be added to the EV2 legend or methods.
Deletion of exon 2 could in principle produce a protein, as the exon is in frame. However, it is unclear whether the first exon would successfully be spliced to the third exon. Furthermore, as the deleted region contains the signal peptide, if any protein is translated it would no longer be targeted to the ER/membrane. If any protein is produced it must be very unstable: based on the antibody staining in FigEV2, no residual protein remains. We have added more explanation about the Δexon2 to the methods and legend.

pg. 6- "expression of genes cut and deadpan" - References?
Thank you for noting the omission, relevant references have been added.

pg. 7- "when they are present on the same surface"- Reference Micchelli et al '97?
Thank you for noting the omission, this reference has been added.

pg. 7- "supressed" - suppressed
We apologise for the mistake, now corrected.

pg. 7 "when combined with a Delta loss of function allele" Mention the data in the figure, especially if this is a new result. If this is an old result, what's the reference?
We have added the figure call-out and also a reference where the result is discussed (de Celis and Bray Development 127, 1291-1302 (2000))

pg. 10- "results coupling C2 domain lipid binding to Notch binding"- What kind of coupling are the authors referring to?
This refers to the observation in Suckling/Korona et al, 2017 that, in a molecular assay of lipid-binding, addition of the Notch11-13 ligand binding fragment to immobilised Jagged-1 was observed to increase the binding of fluorescent liposomes, compared to that seen in the absence of Notch. This suggested that coupling / linking of the two binding events increases the affinity of the complex. We have revised the text to incorporate the original observation and explain the basis for the statement (**page 10 paragraph 2**).

Referee #4

This paper uses structural studies as well as experiments in Drosophila to understand the role of the N-terminal C2 domain of Notch ligands in signalling. It expands on previous work by the authors (Suckling et al. 2017) that indicates the C2 domain is involved in lipid binding.
The document is well written and easy to follow. The paper describes three new crystal structures. These are N-terminal fragments of Drosophila Notch ligands Delta and Serrate, alongside EGFs11-13 of Drosophila Notch, a region known to interact with ligand. As expected, these structures are very similar to their human counterparts. Their in vivo experiments using Drosophila show that deletion of 5 amino acids from a loop in the C2 domain of Delta (located away from the known Notch interaction site) results in phenotypes consistent with disrupted Notch signalling. Although the work does not directly link the C2 domain to phospholipid binding in vivo, it adds further evidence to the hypothesis that that the C2 domain fine-tunes Notch signalling through interactions with the cell membrane.

Commenting specifically, as requested, on evaluation of the structural aspects of the data (Fig. 1, EV1, Table1). The data collection and refinement statistics presented in Table 1 appear sound for all three structures presented (PDB validation reports and examples of electron density were not provided).

I have a few minor comments that would improve the analysis.
-The β1-2 loop in human Delta-like and Jagged ligands is flanked by two cysteines that form a disulphide. These are also present in Delta and Serrate. These have been shown in the figures but not mentioned.
The cysteines are now highlighted in the sequence alignment in **new Figure EV1** and we have added a line to the figure legend to **new Figure EV1** remarking on these.

-Delta is compared to Dll4 and Serrate is compared to Jagged-1. However Dll4 does not have a significant β 1-2 loop, and thus it might be useful to show how Delta compares to Jagged-1 (and Jagged 2) which both have longer loops.
We thank the reviewer for the suggestion, a comparison of the Delta and Serrate structures has been added to **new Figure EV1**

-A sequence alignment highlighting the loops in the C2 domain for Delta/Serrate and human DLL1/DLL4/Jag1/Jag2 proteins would also be a valuable addition.
We thank the reviewer for the suggestion, we have added the sequence alignment to **new Figure EV1**

1st Revision - Editorial Decision 2nd Jul 2021

Dear Prof. Bray

Thank you for the submission of your revised manuscript to EMBO reports. It was sent back to former referee 3 for a final evaluation, who returned a positive report (copied below).

Browsing through the manuscript myself, I noticed a few editorial things that we need before we can proceed with the official acceptance of your study. Please address the following points:

- Please reduce the number of keywords to 5

- Please change the header "Declaration of Interests" to "Conflict of interest".

- Please provide scale bars for Fig. 2B, C, D, Fig. 3A, B, C, E, Fig. EV2B, C, Fig. EV4A-D and define their size in the figure legend.

- Please update the references to the alphabetical Harvard style. The abbreviation 'et al' should be used if more than 10 authors. You can download the respective EndNote file from our Guide to Authors
https://endnote.com/style_download/embo-reports/

- Please correct the following figure callouts in the text:
Fig 2B callout is missing.
Fig 5B+C callouts are missing, but there is a callout to 5D which doesn't exist.
Fig 6 panels are not called out.
Fig EV1, EV3 + EV5 panels are not called out.

- I suggest updating the journal information in the Author checklist.

- During our routine figure analyses we noticed that you use the same thorax microchaetae image for control flies in Fig. 2D and EV4A and for Dl mutant flies in Fig. 2D and EV4C. While this is in principle OK, it is of much more value for the reader to appreciate the variability or reproducibility of mutant phenotypes rather than showing the very same image twice. Therefore, please replace the duplicate images with different ones.

- Table EV1 should be removed from the manuscript file and uploaded individually in word or excel.

- Our production/data editors have asked you to clarify several points in the figure legends (see attached document). Please incorporate these changes in the attached word document and return the revised file with tracked changes with your final manuscript submission. I have also taken the liberty to make some changes to the Abstract. Could you please review it?

- Finally, EMBO reports papers are accompanied online by A) a short (1-2 sentences) summary of the findings and their significance, B) 2-3 bullet points highlighting key results and C) a synopsis image that is 550x200-600 pixels large (width x height) in .png format. You can either show a model or key data in the synopsis image. Please note that the size is rather small and that text needs to be readable at the final size. Please send us this information along with the revised manuscript.

We look forward to seeing a final version of your manuscript as soon as possible.

With kind regards,

Martina Rembold, PhD
Senior Editor
EMBO reports

****

Referee #3:

I think the authors have successfully addressed my original comments, and that the revised manuscript should be accepted.

EMBOR-2021-52729V2: Response to the editor:

We have addressed the points from your e-mail as below. All the changes to the text are made as tracked changes in the manuscript file.

Please address the following points:
- Please reduce the number of keywords to 5.
The number of keywords was reduced to 5.

- Please change the header "Declaration of Interests" to "Conflict of interest".
Changed as requested.

Please provide scale bars for Fig. 2B, C, D, Fig. 3A, B, C, E, Fig. EV2B, C, Fig. EV4A-D and define their size in the figure legend.
Scalebars have been added and defined as requested.

- Please update the references to the alphabetical Harvard style. The abbreviation 'et al' should be used if more than 10 authors.
References have been updated

- Please correct the following figure callouts in the text:
Fig 2B callout is missing.
Fig 5B+C callouts are missing, but there is a callout to 5D which doesn't exist.
Fig 6 panels are not called out.
Fig EV1, EV3 EV5 panels are not called out.
All the call-outs have been corrected as requested.

- I suggest updating the journal information in the Author checklist.
Changed as requested. Manuscript ID added as well.

- During our routine figure analyses we noticed that you use the same thorax microchaetae image for control flies in Fig. 2D and EV4A and for Dl mutant flies in Fig. 2D and EV4C. While this is in principle OK, it is of much more value for the reader to appreciate the variability or reproducibility of mutant phenotypes rather than showing the very same image twice. Therefore, please replace the duplicate images with different ones.
Many thanks for noticing this, we updated the figure EV4 with new images for the control and the Dl mutant flies.

- Table EV1 should be removed from the manuscript file and uploaded individually in word or excel.
Done as requested. Table EV1 was removed from the text and will be uploaded as an individual file.

- Our production/data editors have asked you to clarify several points in the figure legends (see attached document). Please incorporate these changes in the attached word document and return the revised file with tracked changes with your final manuscript submission. I have also taken the liberty to make some changes to the Abstract. Could you please review it?
The points on the figure legends were addressed, including:
- Clarified issues with statistical analysis.
- Added the information about the lines on the violin plots.

- Added the description of the single channel panels.
- Description of the genomic diagram of Dl exon2 allele on figure EV2F.

- Finally, EMBO reports papers are accompanied online by A) a short (1-2 sentences) summary of the findings and their significance, B) 2-3 bullet points highlighting key results and C) a synopsis image that is 550x200-600 pixels large (width x height) in .png format. You can either show a model or key data in the synopsis image. Please note that the size is rather small and that text needs to be readable at the final size. Please send us this information along with the revised manuscript.

Summary, bullet points have been added to the first page of the final version of the manuscript. Synopsis graphic has been uploaded.

Other issues:

We have modified the title along the lines suggested and agree to the suggested changes on the abstract.

2nd Revision - Editorial Decision **15th Jul 2021**

Prof. Sarah J. Bray
Cambridge, University of
Dept. of Physiology, Development and Neuroscience
University of Cambridge
Downing Street
Cambridge,, cambs CB2 3DY
United Kingdom

Dear Prof. Bray,

I am very pleased to accept your manuscript for publication in the next available issue of EMBO reports. Thank you for your contribution to our journal.

At the end of this email I include important information about how to proceed. Please ensure that you take the time to read the information and complete and return the necessary forms to allow us to publish your manuscript as quickly as possible.

As part of the EMBO publication's Transparent Editorial Process, EMBO reports publishes online a Review Process File to accompany accepted manuscripts. As you are aware, this File will be published in conjunction with your paper and will include the referee reports, your point-by-point response and all pertinent correspondence relating to the manuscript.

If you do NOT want this File to be published, please inform the editorial office within 2 days, if you have not done so already, otherwise the File will be published by default [contact: emboreports@embo.org]. If you do opt out, the Review Process File link will point to the following statement: "No Review Process File is available with this article, as the authors have chosen not to make the review process public in this case."

Should you be planning a Press Release on your article, please get in contact with emboreports@wiley.com as early as possible, in order to coordinate publication and release dates.

Thank you again for your contribution to EMBO reports and congratulations on a successful publication. Please consider us again in the future for your most exciting work.

Yours sincerely,

Achim Breiling
Editor
EMBO Reports

****

THINGS TO DO NOW:

You will receive proofs by e-mail approximately 2-3 weeks after all relevant files have been sent to our Production Office; you should return your corrections within 2 days of receiving the proofs.

Please inform us if there is likely to be any difficulty in reaching you at the above address at that time. Failure to meet our deadlines may result in a delay of publication, or publication without your corrections.

All further communications concerning your paper should quote reference number EMBOR-2021-52729V3 and be addressed to emboreports@wiley.com.

Should you be planning a Press Release on your article, please get in contact with emboreports@wiley.com as early as possible, in order to coordinate publication and release dates.

EMBO PRESS

YOU MUST COMPLETE ALL CELLS WITH A PINK BACKGROUND ➔

PLEASE NOTE THAT THIS CHECKLIST WILL BE PUBLISHED ALONGSIDE YOUR PAPER

Corresponding Author Name: Penny Handford, Susan M. Lea and Sarah Bray
Journal Submitted to: EMBO Reports
Manuscript Number: EMBOR-2021-52729V2

Reporting Checklist For Life Sciences Articles (Rev. June 2017)

This checklist is used to ensure good reporting standards and to improve the reproducibility of published results. These guidelines are consistent with the Principles and Guidelines for Reporting Preclinical Research issued by the NIH in 2014. Please follow the journal's authorship guidelines in preparing your manuscript.

A- Figures
1. Data
The data shown in figures should satisfy the following conditions:

- the data were obtained and processed according to the field's best practice and are presented to reflect the results of the experiments in an accurate and unbiased manner.
- figure panels include only data points, measurements or observations that can be compared to each other in a scientifically meaningful way.
- graphs include clearly labeled error bars for independent experiments and sample sizes. Unless justified, error bars should not be shown for technical replicates.
- if n< 5, the individual data points from each experiment should be plotted and any statistical test employed should be justified
- Source Data should be included to report the data underlying graphs. Please follow the guidelines set out in the author ship guidelines on Data Presentation.

2. Captions

Each figure caption should contain the following information, for each panel where they are relevant:

- a specification of the experimental system investigated (eg cell line, species name).
- the assay(s) and method(s) used to carry out the reported observations and measurements
- an explicit mention of the biological and chemical entity(ies) that are being measured.
- an explicit mention of the biological and chemical entity(ies) that are altered/varied/perturbed in a controlled manner.

- the exact sample size (n) for each experimental group/condition, given as a number, not a range;
- a description of the sample collection allowing the reader to understand whether the samples represent technical or biological replicates (including how many animals, litters, cultures, etc.).
- a statement of how many times the experiment shown was independently replicated in the laboratory.
- definitions of statistical methods and measures:
  - common tests, such as t-test (please specify whether paired vs. unpaired), simple χ2 tests, Wilcoxon and Mann-Whitney tests, can be unambiguously identified by name only, but more complex techniques should be described in the methods section;
  - are tests one-sided or two-sided?
  - are there adjustments for multiple comparisons?
  - exact statistical test results, e.g., P values = x but not P values < x;
  - definition of 'center values' as median or average;
  - definition of error bars as s.d. or s.e.m.

Any descriptions too long for the figure legend should be included in the methods section and/or with the source data.

In the pink boxes below, please ensure that the answers to the following questions are reported in the manuscript itself. Every question should be answered. If the question is not relevant to your research, please write NA (non applicable). We encourage you to include a specific subsection in the methods section for statistics, reagents, animal models and human subjects.

USEFUL LINKS FOR COMPLETING THIS FORM

http://www.antibodypedia.com
http://1degreebio.org
http://www.equator-network.org/reporting-guidelines/improving-bioscience-research-repor
http://grants.nih.gov/grants/olaw/olaw.htm
http://www.mrc.ac.uk/Ourresearch/Ethicsresearchguidance/Useofanimals/index.htm
http://ClinicalTrials.gov
http://www.consort-statement.org
http://www.consort-statement.org/checklists/view/32-consort/66-title
http://www.equator-network.org/reporting-guidelines/reporting-recommendations-for-tumo
http://datadryad.org
http://figshare.com
http://www.ncbi.nlm.nih.gov/gap
http://www.ebi.ac.uk/ega
http://biomodels.net/
http://biomodels.net/miriam/
http://jjj.biochem.sun.ac.za
http://oba.od.nih.gov/biosecurity/biosecurity_documents.html
http://www.selectagents.gov/

B- Statistics and general methods

Please fill out these boxes ➔ (Do not worry if you cannot see all your text once you press return)

1.a. How was the sample size chosen to ensure adequate power to detect a pre-specified effect size?	No power analysis was used. In each case comparisons were made between distinct genotypes and differences were detected in samples and images collected on multiple days. Quantifications were used to exemplify the levels observed by eye. Data were only incuded when the results were reproducible between replicates and sufficient replicates performed for the results to be convincing with the aid of statistical tests.
1.b. For animal studies, include a statement about sample size estimate even if no statistical methods were used.	The number of biological replicates is given for every experiment in the figure legends. All the measurements are represented as dot-plots, with each point measurements from a different fly/larva.
2. Describe inclusion/exclusion criteria if samples or animals were excluded from the analysis. Were the criteria pre-established?	NA:No samples or animals were excluded from analysis.
3. Were any steps taken to minimize the effects of subjective bias when allocating animals/samples to treatment (e.g. randomization procedure)? If yes, please describe.	NA: animals/samples were not allocated to different treatments in our study. All genotypes and samples were treated equally and comparisons made between them. When comparing different genotypes and assaying for genetic interactions, all the crosses were performed in parallel to ensure they were all exposed to the same conditions.
For animal studies, include a statement about randomization even if no randomization was used.	NA: no animal experiment involved allocating different treatments.
4.a. Were any steps taken to minimize the effects of subjective bias during group allocation or/and when assessing results (e.g. blinding of the investigator)? If yes please describe.	No allocations to groups was required, samples analyzed were distinct genotypes. The investigator was not blinded. However, all samples were treated equally and strict criteria were used to assess the phenotypes.
4.b. For animal studies, include a statement about blinding even if no blinding was done	All experiments involved mutiple replicates amd strict criteria were used to compare genotypes. In several experiments, the tissue analyzed was a mosaic of wild-type and mutant tissue enabling direct comparisons in the same animal. No blinding was done.
5. For every figure, are statistical tests justified as appropriate?	Yes. Statistical tests are noted in every case and the tests used were standard ones. All differences were of a magnitude that was already obvious by eye.
Do the data meet the assumptions of the tests (e.g., normal distribution)? Describe any methods used to assess it.	The assumptions of independent and random sampling are valid for all experiments. No formal methods were used to assess normality or equality of variance. However, student's t-tests (equal variance) were used only for experiments where the scatter of data did not overtly change between samples and theoretically should not have changed.

Is there an estimate of variation within each group of data?	Standard error of the mean is shown in all graphs.
Is the variance similar between the groups that are being statistically compared?	Yes, variance was similar between groups.

C- Reagents

6. To show that antibodies were profiled for use in the system under study (assay and species), provide a citation, catalog number and/or clone number, supplementary information or reference to an antibody validation profile. e.g., Antibodypedia (see link list at top right), 1DegreeBio (see link list at top right).	References for use and validation of antibodies are provided in the Material and Methods. All are from well-established sources and have been used by multiple investigators in the field
7. Identify the source of cell lines and report if they were recently authenticated (e.g., by STR profiling) and tested for mycoplasma contamination.	No cell lines were used.

* for all hyperlinks, please see the table at the top right of the document

D- Animal Models

8. Report species, strain, gender, age of animals and genetic modification status where applicable. Please detail housing and husbandry conditions and the source of animals.	The specie used in this study was the Drosophila melanogaster. Strains and origins are given in the Methods section. All stocks are kept in a dedicated facility complying with regulations.
9. For experiments involving live vertebrates, include a statement of compliance with ethical regulations and identify the committee(s) approving the experiments.	NA: no vertebrate models used.
10. We recommend consulting the ARRIVE guidelines (see link list at top right) (PLoS Biol. 8(6), e1000412, 2010) to ensure that other relevant aspects of animal studies are adequately reported. See author guidelines, under 'Reporting Guidelines'. See also: NIH (see link list at top right) and MRC (see link list at top right) recommendations. Please confirm compliance.	We comply with the ARRIVE guidelines.

E- Human Subjects

11. Identify the committee(s) approving the study protocol.	NA: no human subjects.
12. Include a statement confirming that informed consent was obtained from all subjects and that the experiments conformed to the principles set out in the WMA Declaration of Helsinki and the Department of Health and Human Services Belmont Report.	NA: no human subjects.
13. For publication of patient photos, include a statement confirming that consent to publish was obtained.	NA: no human subjects.
14. Report any restrictions on the availability (and/or on the use) of human data or samples.	NA: no human subjects.
15. Report the clinical trial registration number (at ClinicalTrials.gov or equivalent), where applicable.	NA: no human subjects.
16. For phase II and III randomized controlled trials, please refer to the CONSORT flow diagram (see link list at top right) and submit the CONSORT checklist (see link list at top right) with your submission. See author guidelines, under 'Reporting Guidelines'. Please confirm you have submitted this list.	NA: no human subjects.
17. For tumor marker prognostic studies, we recommend that you follow the REMARK reporting guidelines (see link list at top right). See author guidelines, under 'Reporting Guidelines'. Please confirm you have followed these guidelines.	NA: no human subjects.

F- Data Accessibility

18: Provide a "Data Availability" section at the end of the Materials & Methods, listing the accession codes for data generated in this study and deposited in a public database (e.g. RNA-Seq data: Gene Expression Omnibus GSE39462, Proteomics data: PRIDE PXD000208 etc.) Please refer to our author guidelines for 'Data Deposition'.<br><br>Data deposition in a public repository is mandatory for:<br>a. Protein, DNA and RNA sequences<br>b. Macromolecular structures<br>c. Crystallographic data for small molecules<br>d. Functional genomics data<br>e. Proteomics and molecular interactions	Protein structures have been deposited in a public repository and the accession numbers are provided in the Methods
19. Deposition is strongly recommended for any datasets that are central and integral to the study; please consider the journal's data policy. If no structured public repository exists for a given data type, we encourage the provision of datasets in the manuscript as a Supplementary Document (see author guidelines under 'Expanded View' or in unstructured repositories such as Dryad (see link list at top right) or Figshare (see link list at top right).	NA: no largescale data sets have been generated or used.
20. Access to human clinical and genomic datasets should be provided with as few restrictions as possible while respecting ethical obligations to the patients and relevant medical and legal issues. If practically possible and compatible with the individual consent agreement used in the study, such data should be deposited in one of the major public access-controlled repositories such as dbGAP (see link list at top right) or EGA (see link list at top right).	NA: No human clinical data has been used.
21. Computational models that are central and integral to a study should be shared without restrictions and provided in a machine-readable form. The relevant accession numbers or links should be provided. When possible, standardized format (SBML, CellML) should be used instead of scripts (e.g. MATLAB). Authors are strongly encouraged to follow the MIRIAM guidelines (see link list at top right) and deposit their model in a public database such as Biomodels (see link list at top right) or JWS Online (see link list at top right). If computer source code is provided with the paper, it should be deposited in a public repository or included in supplementary information.	NA: No computer models have been used.

G- Dual use research of concern

22. Could your study fall under dual use research restrictions? Please check biosecurity documents (see link list at top right) and list of select agents and toxins (APHIS/CDC) (see link list at top right). According to our biosecurity guidelines, provide a statement only if it could.	No, our study does not fall under dual use research restrictions.
